# Individualized Prediction of Transition to Psychosis in 1,676 Individuals at Clinical High Risk: Development and Validation of a Multivariable Prediction Model Based on Individual Patient Data Meta-Analysis

**DOI:** 10.3389/fpsyt.2019.00345

**Published:** 2019-05-21

**Authors:** Aaltsje Malda, Nynke Boonstra, Hans Barf, Steven de Jong, Andre Aleman, Jean Addington, Marita Pruessner, Dorien Nieman, Lieuwe de Haan, Anthony Morrison, Anita Riecher-Rössler, Erich Studerus, Stephan Ruhrmann, Frauke Schultze-Lutter, Suk Kyoon An, Shinsuke Koike, Kiyoto Kasai, Barnaby Nelson, Patrick McGorry, Stephen Wood, Ashleigh Lin, Alison Y. Yung, Magdalena Kotlicka-Antczak, Marco Armando, Stefano Vicari, Masahiro Katsura, Kazunori Matsumoto, Sarah Durston, Tim Ziermans, Lex Wunderink, Helga Ising, Mark van der Gaag, Paolo Fusar-Poli, Gerdina Hendrika Maria Pijnenborg

**Affiliations:** ^1^GGZ Friesland Mental Health Institute, Leeuwarden, Netherlands; ^2^University of Groningen, Groningen, Netherlands; ^3^NHL Stenden University of Applied Sciences, Leeuwarden, Netherlands; ^4^Lentis Psychiatric Institute, Groningen, Netherlands; ^5^Cognitive Neuroscience Center, Department of Biomedical Sciences of Cells and Systems, University Medical Center Groningen, Groningen, Netherlands; ^6^Department of Psychiatry, Hotchkiss Brain Institute, University of Calgary, Calgary, Alberta, Canada; ^7^Prevention and Early Intervention Program for Psychosis, Douglas Mental Health University Institute, McGill University, Montreal, QC, Canada; ^8^Department of Psychology, University of Konstanz, Konstanz, Germany; ^9^Amsterdam University Medical Centers, Location AMC, Department of Psychiatry, Amsterdam, Netherlands; ^10^Division of Psychology and Mental Health, University of Manchester, Manchester, United Kingdom; ^11^Psychosis Research Unit, Greater Manchester Mental Health NHS Foundation Trust, Manchester, United Kingdom; ^12^University of Basel Psychiatric Hospital, Basel, Switzerland; ^13^Department of Psychiatry and Psychotherapy, University of Cologne, Cologne, Germany; ^14^Department of Psychiatry and Psychotherapy, Medical Faculty, Heinrich-Heine University, Düsseldorf, Germany; ^15^Department of Psychiatry, Severance Hospital, Yonsei University College of Medicine, Seoul, South Korea; ^16^University of Tokyo Institute for Diversity and Adaptation of Human Mind (UTIDAHM), Tokyo, Japan; ^17^Department of Neuropsychiatry, Graduate School of Medicine, The University of Tokyo, Tokyo, Japan; ^18^Tokyo Center for Integrative Science of Human Behaviour (CiSHuB), The University of Tokyo, Tokyo, Japan; ^19^The International Research Center for Neurointelligence (WPI-IRCN) at The University of Tokyo Institutes for Advanced Study (UTIAS), The University of Tokyo, Tokyo, Japan; ^20^Orygen, The National Centre of Excellence in Youth Mental Health, Melbourne, VIC, Australia; ^21^Centre for Youth Mental Health, The University of Melbourne, Melbourne, VIC, Australia; ^22^School of Psychology, University of Birmingham, Birmingham, United Kingdom; ^23^Telethon Kids Institute, The University of Western Australia, Perth, WA, Australia; ^24^Greater Manchester Mental Health NHS Foundation Trust, Manchester, United Kingdom; ^25^Faculty of Biology, Medicine and Health, University of Manchester, Manchester, United Kingdom; ^26^Department of Affective and Psychotic Disorders, Medical University of Lodz, Lodz, Poland; ^27^Child and Adolescence Neuropsychiatry Unit, Department of Neuroscience, Children Hospital Bambino Gesù, Rome, Italy; ^28^Office Médico-Pédagogique Research Unit, Department of Psychiatry, University of Geneva, School of Medicine, Geneva, Switzerland; ^29^Department of Psychiatry, Tohoku University Hospital, Sendai, Japan; ^30^Department of Psychiatry, Tohoku University Graduate School of Medicine, Sendai, Japan; ^31^Department of Preventive Psychiatry, Tohoku University Graduate School of Medicine, Sendai, Japan; ^32^NICHE Lab, Department of Psychiatry, Brain Center Rudolf Magnus, University Medical Center, Utrecht, Netherlands; ^33^Department of Psychology, University of Amsterdam, Amsterdam, Netherlands; ^34^University Medical Center Groningen, Groningen, Netherlands; ^35^Department of Clinical Psychology, VU University, Amsterdam, Netherlands; ^36^Parnassia Psychiatric Institute, Department of Psychosis Research, Den Haag, Netherlands; ^37^Early Psychosis: Interventions and Clinical-detection (EPIC) lab, Department of Psychosis Studies, Institute of Psychiatry, Psychology and Neuroscience, King’s College London, London, United Kingdom; ^38^OASIS Service, South London and Maudsley NHS Foundation Trust, London, United Kingdom; ^39^Department of Brain and Behavioral Sciences, University of Pavia, Pavia, Italy; ^40^National Institute for Health Research, Biomedical Research Centre for Mental Health, South London and Maudsley NHS Foundation Trust, London, United Kingdom; ^41^GGZ Drenthe Mental Health Care Center, Department of Psychotic Disorders, Assen, Netherlands

**Keywords:** clinical high risk, psychosis, schizophrenia, individual patient data meta-analysis, prognosis, risk prediction

## Abstract

**Background:** The Clinical High Risk state for Psychosis (CHR-P) has become the cornerstone of modern preventive psychiatry. The next stage of clinical advancements rests on the ability to formulate a more accurate prognostic estimate at the individual subject level. Individual Participant Data Meta-Analyses (IPD-MA) are robust evidence synthesis methods that can also offer powerful approaches to the development and validation of personalized prognostic models. The aim of the study was to develop and validate an individualized, clinically based prognostic model for forecasting transition to psychosis from a CHR-P stage.

**Methods:** A literature search was performed between January 30, 2016, and February 6, 2016, consulting PubMed, Psychinfo, Picarta, Embase, and ISI Web of Science, using search terms (“ultra high risk” OR “clinical high risk” OR “at risk mental state”) AND [(conver* OR transition* OR onset OR emerg* OR develop*) AND psychosis] for both longitudinal and intervention CHR-P studies. Clinical knowledge was used to *a priori* select predictors: age, gender, CHR-P subgroup, the severity of attenuated positive psychotic symptoms, the severity of attenuated negative psychotic symptoms, and level of functioning at baseline. The model, thus, developed was validated with an extended form of internal validation.

**Results:** Fifteen of the 43 studies identified agreed to share IPD, for a total sample size of 1,676. There was a high level of heterogeneity between the CHR-P studies with regard to inclusion criteria, type of assessment instruments, transition criteria, preventive treatment offered. The internally validated prognostic performance of the model was higher than chance but only moderate [Harrell’s C-statistic 0.655, 95% confidence interval (CIs), 0.627–0.682].

**Conclusion:** This is the first IPD-MA conducted in the largest samples of CHR-P ever collected to date. An individualized prognostic model based on clinical predictors available in clinical routine was developed and internally validated, reaching only moderate prognostic performance. Although personalized risk prediction is of great value in the clinical practice, future developments are essential, including the refinement of the prognostic model and its external validation. However, because of the current high diagnostic, prognostic, and therapeutic heterogeneity of CHR-P studies, IPD-MAs in this population may have an limited intrinsic power to deliver robust prognostic models.

## Introduction

Clinical research on early recognition and intervention of psychotic disorders has enormously expanded over the past two decades (1). There is converging evidence that individuals with an elevated risk for psychosis, commonly termed as at Clinical Risk for Psychosis [CHR-P; or as “ultra high risk” (UHR) or “at‐risk mental state” (ARMS)], can be identified prior to the onset of a psychotic episode. CHR-P criteria are based by the presence of attenuated psychotic symptoms, brief and intermittent psychotic symptoms with spontaneous remission, or genetic risk for psychosis (2–4), usually combined with functional impairments and help-seeking behavior (5). CHR-P individuals accumulate several risk factors for psychosis (6) and have a meta-analytical risk of developing psychosis of 20% [95% confidence interval (95% CI) 17%–25%] at 2 years [for details, see Table 4 in Fusar-Poli et al. (7)] while they are not an increased risk for developing non-psychotic mental disorders (8). The level of risk for psychosis is highest in those with a short-lived psychotic episode, intermediate in those with attenuated positive psychotic symptoms and lowest in those at genetic risk (9). Overall, the meta-analytical prognostic performance of the CHR-P assessment is excellent [area under the curve (AUC) of 0.9 at 38 months] (10) and comparable to that of prognostic models used in other branches of somatic medicine. Despite these achievements, to date, the formulation of a prognosis in CHR-P individuals has been limited to group-level predictions. In light of the recent emergence of precision medicine approaches, it is thus important to develop and validate prognostic models that can calculate a personalized risk rather than a group-level global risk estimate. Prognostic modeling combines multiple predictor variables with their relative weight to estimate the risk or probability that an outcome or specific event will occur in an individual patient (11) and is often used in medical sciences, such as cardiology or oncology [e.g., Refs. (12, 13)]. The calculated individual risks could then be utilized by the caregiver to inform treatment decisions.

More recently, prognostic models have entered clinical psychiatry [for a methodological review, see Fusar-Poli et al. (14)]. A systematic review has identified seven prognostic models for CHR-P populations, most of which suffer from methodological weaknesses, such as the use of suboptimal model building methods, small sample sizes, and the lack of internal or external validation (15). Several recommendations for building robust prognostic models in CHR-P populations were made, including the use of large sample sizes, appropriate events per variable ratios, the selection of *a priori* predictors on the basis of clinical knowledge or the use of automated selection features through machine-learning methods, and the essential need to present validated (internal and external) measures of prognostic performance (14). Some examples of robust prognostic subject-level models for CHR-P populations include the nothern american prodrome longitudinal study (NAPLS) risk calculator by Cannon et al. (16) [which has been externally validated (17)], the pretest risk enrichment stratification algorithm by Fusar-Poli et al. (18) (which has been externally validated), the transdiagnostic risk calculator by Fusar-Poli et al. (19) [which has been externally validated twice (20) and implemented in clinical routine (21)], and the functional outcome prognostic model by Koutsouleris et al. (22) (internally validated). Yet, the key create-limiting step toward implementation of prognostic models into CHR-P clinical routine is the availability of predictors. Biological and neurophysiological data require more expensive and intrusive assessment methods which are not always available in clinical practice, limiting the clinical utility of these models. Rather, neurobiological-based prognostic models can further refine the prediction of outcomes when used in a stepped sequential framework (23), after simpler prognostic models are applied.

We present here an innovative approach for developing risk prediction models for CHR-P individuals that are based on clinical predictors routinely collected as part of clinical practice. We developed a multivariable (i.e., including several predictors) risk estimation model through re-analyzing original individual raw data, requested from systemically sought research groups (24), through an individual patient data meta-analysis (IPD-MA). Prognostic models developed from an IPD-MA offer several unexplored advantages, such as large sample sizes, which are of core importance in the case of rare events, such as the transition to psychosis from CHR-P stage (25). Moreover, because an IPD-MA leverages the variation in the characteristics of the CHR-P included, it can potentially increase the generalizability of the prognostic model. Furthermore, a prognostic model derived from IPD-MA can statistically take into account the differences in prognostic parameters (such as intercepts and predictor-outcome associations) across the included original studies and can explore under which circumstances the prognostic model predicts optimally (26). Despite these potentials, no IPD-MA has ever been conducted in the CHR-P field.

The primary aim of the current study was to develop and validate an individualized, clinically based prognostic model for forecasting transition to psychosis from a CHR-P stage using predictors that were selected on the basis of *a priori* clinical knowledge and that were available in clinical routine.

## Methods

### Search Strategies

A systematic search strategy was performed to identify relevant original studies. First, an electronic search was performed in PubMed, Psychinfo, Picarta, Embase, and ISI Web of Science. The search was conducted between January 30, 2016, and February 6, 2016. The following search terms were used: (“ultra high risk” OR “clinical high risk” OR “at risk mental state”) AND [(conver* OR transition* OR onset OR emerg* OR develop*) AND psychosis]. Second, the reference lists of the included articles were manually checked for studies not identified by the computerized search.

### Selection Criteria

Inclusion criteria were as follows:

1. data reported in an original paper in a peer-reviewed journal;
2. involved CHR-P subjects 14 to 40 years old, defined according to established international criteria (1);
3. assessed attenuated positive and negative psychotic symptoms as well as level of functioning at baseline using standardized CHR-P measurements;
4. reported transition status at follow-up (events);
5. reported time to transition or time to last follow-up assessment.

Both longitudinal and intervention studies were included. In the case of studies investigating heterogeneous patient populations, only CHR-P individuals were selected for the analysis. Furthermore, CHR-P individuals who were not meeting the age criterion defined above were excluded from the analysis, as well as CHR-P patients who were already psychotic at baseline as documented in the corresponding articles.

To achieve a high standard of reporting, we adopted the Preferred Reporting Items for Systematic Reviews and Meta-analyses Guidelines-Individual Patient Data (PRISMA-IPD), (27) as well as the statement transparent reporting of a multivariable prediction model for individual prognosis or diagnosis (TRIPOD) (28). The meta-analysis was registered in the PROSPERO database for systematic reviews and meta-analysis (CRD42017071176).

### Selection of Predictors

For developing and validating a prediction model, it is recommended to select prognostic variables *a priori* based on earlier research (28) and clinical knowledge (14). To develop a model that is readily applicable in clinical practice, the selected predictors were limited to those routinely assessed in CHR-P clinics and involved demographical and clinical predictors. The *a priori* selected predictors were age, gender, CHR-P subgroup (attenuated psychotic symptoms, brief and limited intermittent psychotic symptoms, genetic risk, and deterioration syndrome), baseline severity of attenuated positive and negative psychotic symptoms, and level of functioning. The *a priori* clinical rationale for selecting these predictors is given below. The first predictor is age: in general, youth in their late teens and early 20s have the highest risk of developing psychosis (29) and a meta-analysis revealed that older CHR-P individuals had a significant higher risk for developing a psychotic episode (30). Another recent umbrella review found that those aged 15 to 35 years have a strong factor associated with an increased risk of psychosis (31). The same umbrella review found that gender, the second predictor in our model, has a clear association with an increased risk of psychosis (31). In fact, gender has already been used as predictor in other prognostic models developed for CHR-P populations (19). The third predictor was the severity of attenuated positive psychotic symptoms, such as delusions, unusual thought content, and suspicion, which are the most studied and established predictors in CHR-P field and already used by previous prognostic tools in this group (16). Furthermore, a recent meta-analysis of 33 studies, involving a total of 4,227 CHR-P individuals, showed different levels of the risk for psychosis onset, where persons with brief and limited intermittent psychotic symptoms had the highest risk of transition, followed by those with attenuated positive psychotic symptoms, and by those with genetic risk and deterioration syndrome who had the lowest risk (9). Therefore, the CHR-P subgroups were included as three independent predictors, recording whether or not the criteria of each distinctive risk group were met. Attenuated negative psychotic symptoms encompass social amotivation (apathy, anhedonia, asociality) and expressive deficits (alogia, diminished emotional expression) (32) and were selected as the seventh predictor. Attenuated negative psychotic symptoms were predictive of a subsequent psychotic disorder in CHR-P individuals (33, 34). The last predictor variable was the level of functioning at baseline: a meta-analysis in CHR-P individuals confirmed that functioning is a strong predictor of transition to psychosis (35).

### Data Collection

Abstracts were screened independently by two reviewers (AM and NB or MP). Each article was assessed individually, and any disagreements resolved by discussion with a third reviewer. Subsequently, all corresponding authors of the eligible studies identified were contacted to request anonymized individual patient data and regarded as non-responders when no reaction was received after two reminder emails.

### Data Extraction

From each individual patient, the following variables were included: gender, the baseline age of participant, CHR-P group, the severity of attenuated psychotic positive and negative symptoms, level of functioning, transition status at follow-up, and duration of the follow-up period. To get a better understanding of possible factors that may have influenced the performance of the prognostic model across the different studies, as well as to detect factors that may have contributed to the study heterogeneity, we also collected for each study additional data. These data are related to the inclusion period, inclusion strategies, inclusion and exclusion criteria, the psychometric criteria employed to define transition to psychosis and the type of CHR-P assessment instruments [for a comparative analysis of CHR-P assessment instruments, see Fusar-Poli et al. (36)], and the instruments applied to assess symptoms and functioning.

### Data Storage

All data were anonymized by the researchers of the original studies and therefore not re-identifiable to an individual patient by the current investigators. All cleaned data sets were stored on a secured server in their original formats and converted to a master data set. Data were inspected on unusual outliers *via* range check of the all included variables.

### Data Transformation

Studies vary in the CHR-P instruments assessing the severity of attenuate positive psychotic symptoms, attenuated negative psychotic symptoms, and functioning. Thus, to make it clinically applicable, only one measurement was selected in the model as the primary parameter. The selection of the assessment measure was defined *a priori* on the basis of clinical reasoning.

### Missing Data

Missing data were imputed according to Multiple Imputations with Chained Equations (MICE) with 50 iterations sets. As recommended by White and Royston (37), the event indicator and Nelson-Aalen estimator of cumulative baseline hazard were included in the imputation model. Also, the study name of the original data was included as a dummy factor to account for potential between-study heterogeneity. Rubin’s Rules were applied to combine the data from the imputation sets (38).

### Risk of Bias Assessment in Individual Studies

The assessment of the methodological quality of each individual included study is an essential element in meta-analyses (27). The majority of the studies in this IPD-MA have a naturalistic observational design (N = 12). As such, we used the systematic review of Zeng et al. (39), which recommends the Newcastle-Ottawa Scale (NOS) (40), a nine-item scale categorized into three dimensions, namely, selection, comparability, and outcome. Quality assessment of naturalistic and observational studies in meta-analyses is problematic. In fact, the key components of studies to be assessed on the MOOSE’s recommendations were whether the outcome of interest was not present at the start of the study, the follow-up period of the study was long enough for the outcome to occur, and an adequate proportion of the subjects participated in the follow-up cohort (41). The minimal follow-up period in this IPD-MA was set at 12 months. Studies received a positive score for adequacy of follow-up cohort when they had a minimum follow-up rate of 50% to 80% in cohort studies or 80% in randomized controlled trials (RCTs) (42).

### Primary Outcome

The primary outcome is the transition to psychosis (event) from a CHR-P stage. Transition to psychosis was defined according to the criteria of the Comprehensive Assessment of At Risk Mental State (CAARMS) (2), Structured Interview of Prodromal Symptoms/Scale of Prodromal Symptoms (SIPS/SOPS) (3), Brief Psychiatric Rating Scale (BPRS) (43), Positive and Negative Syndrome Scale (PANSS) (44), or Structured Interview for Diagnostic and Statistical Manual of Mental Disorders, fourth edition (SCID-IV) (45). The CHR-P patient outcomes were recorded as transitioned to a psychosis, no transition, or lost to follow-up.

### Data Analyses

Individuals with a complete follow-up assessment were compared with those lost to follow-up with an independent *t* test (continuous variables) or chi-square test (binary variables) for descriptive purposes. Collinearity of predictors was tested with the variance inflation factor (VIF) and estimated by the formula 1/(1 − *R* ^2^). An outcome of 4 or lower indicates a low indication of collinearity between the predictors (46).

A parametric survival model with a log-normal distribution for event times was computed (47). The evaluation of the model’s performance and generalizability was done with an extended form of internal validation, because of the lack of true external validation data. Therefore, an internal–external cross validation (IECV) technique was applied, which maximized the data available for both model development as well as model validation (26). With the IECV, all studies (*M*) minus one study were used as a derivation set to develop a prediction model, and the remaining set is used for its external validation. This was repeated for each data set, leading to *M* scenarios to investigate consistent model performance, which was combined by applying Rubin’s Rules (38). All discovered studies were utilized in the development and validation of the model. A *t* test calculated the significance of the final beta coefficients of the predictors.

The model performance was estimated by calculating its discrimination and calibration. Discrimination referred to the model’s ability to separate CHR-P individuals who transitioned to psychosis versus those who did not transition. For each study, a bar graph with the frequency distribution of predicted survival of the survival groups was presented, for both 12 months as well as 24 months. For both 12 and 24 months, the bar graph showed 10 risk groups, which each represented an equal number of individuals. The distribution of the risk groups, which ranged from 0 (no chance of survival, i.e. transition to a psychosis) until 100 (100% chance of survival, so no transition to psychosis) was determined by the observed survival per study. A well-discriminating model shows a high overlap between the predicted survival and the observed survival in the different risk categories (48). Moreover, Harrell’s C statistics with its 95% CI was calculated per study, which referred to the overall probability that the model estimates a higher risk for the CHR-P individual that does develop psychosis compared with a person that does not. Values of C-statistics higher than 0.5 (random prediction) and lower than 0.6 are considered “poor”; from 0.6 and 0.7 are considered “moderate”; from 0.7 to 0.8, “adequate”; from 0.8 to 0.9, “excellent”; and above 0.9, “outstanding,” up to 1 (perfect prediction). The C-statistics of all individual studies was plotted in a forest plot, with the 95% CI indicating a possible statistical difference from random prediction. Furthermore, for each study, the calibration of the model was calculated, which referred to the agreement between the observed and the predicted outcomes (48) and was presented with its 95% CI for each individual article in a forest plot. The linear predictor is calculated according to the coefficients of the model and included as a covariate in a Cox model. The slope of the linear predictor is the calibration slope. The calibration plot can be viewed as a measure of fit of the prognostic model in the CHR-P population: when a study’s 95% CI included the value of 1, it indicated a fit, whereas a 95% CI not containing a score of 1 implied a serious misfit of the model, suggesting that adjustments of the model’s intercepts should be considered.

The CHR-P studies differed with regard to study design, inclusion period, recruitment strategies, inclusion and exclusion criteria, transition criteria, CHR-P assessment instruments, and treatments offered. These characteristics were expected to influence the effects of the prognostic model in this IPD-MA. In meta-analyses, heterogeneity is examined with the Q-statistic and I^2^ Index (24). However, in studies that develop prediction models based on IPD-MA, the extent of heterogeneity is better quantified by studying the 95% prediction intervals (49).

All statistical analyses were conducted using R version 4.2.2 (50) and used the following packages: foreign, mice, micemd, Hmsic, VIM, jomo, flexsurv, metamisc, rms, and pec.

## Results

### Studies and Participants

**Figure 1:**
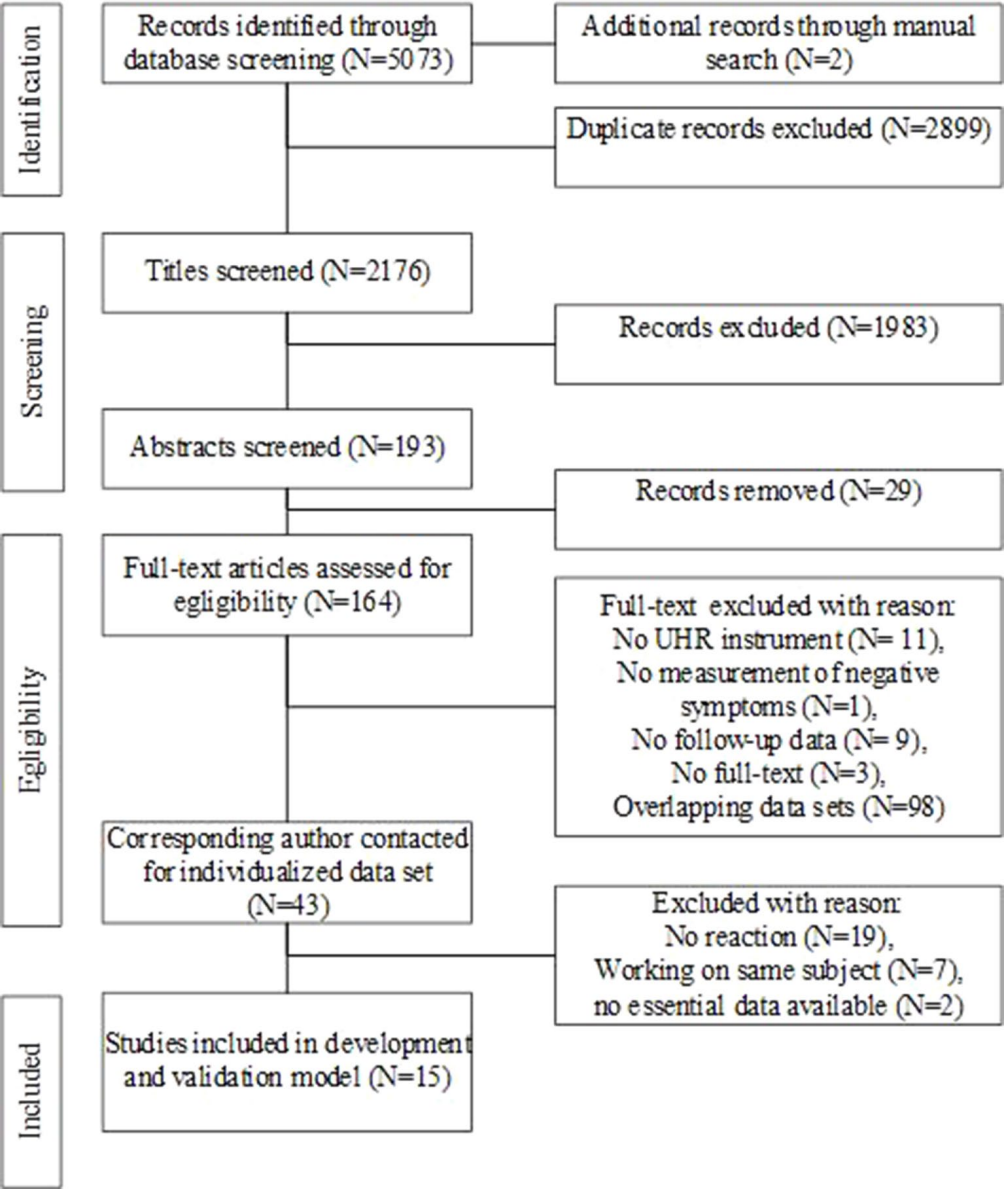
Flow chart of the study.

A total of 2,176 papers were identified by the literature search and 43 were deemed eligible for the IPD-MA. The corresponding authors of the 43 studies were contacted, of which 15 agreed to participate and shared all necessary individual patient data needed for the model (see **Figure 1**). Of the remaining authors, seven authors replied to work on the same subject, two were not able to share the essential data, and nineteen authors did not reply at all. These 28 studies related to a total of 2,815 CHR-P individuals (62.7% of CHR-P eligible subjects), of whom 475 transitioned to psychosis (16.9% of the eligible yet not included subjects). There is a selection bias in that the current IPD-MA included 1,676 CHR-P individuals, of whom 386 developed psychosis. This corresponded to 37.3% of all the CHR-P eligible participants.

The participating studies were Access, Detection And Psychosocial Treatment (ADAPT) (51), Clinic for Assessment of Youth at Risk (CAYR) (52), Dutch Prediction of Psychosis Study-Amsterdam (DUPS-A) (53), Early Detection and Intervention Evaluation-Netherlands (EDIE-NL) (54), Early Detection and Intervention-United Kingdom (EDIE-UK) (55), Früherkennung von Psychosen (FePsy) (56), Früherkennungs- und Therapiezentrum für psychische Krisen (FETZ) (57), Green Program for Recognition and Prevention of Early Psychosis (GRAPE) (58), Integrative Neuroimaging Studies in Schizophrenia Targeting for Early intervention and Prevention (IN-STEP) (59), Outreach and Support in South London (OASIS) (60), Personal Assessment and Crisis Evaluation (PACE) (61), Programme of Recognition and Therapy (PORT) (62), ROME (63), Sendai ARMS and First Episode clinic (SAFE) (64), and Dutch Prediction of Psychosis Study-Utrecht (DUPS-U) (65).

Furthermore, for each included study, we checked whether CHR-P individuals met the inclusion criteria. CHR-P individuals younger than 14 years were removed from the data set: ADAPT (N = 2), CAYR (N = 1), DUPS (N = 4), EDIE-NL (N = 1), PACE (N = 1), Rome (N = 19), and DUPS-U (N = 14), as well as participants older than 40 years: FePsy (N = 10) and IN-STEP (N = 1). Subjects with an elevated risk for psychosis but not meeting the established CHR criteria were excluded: FePsy (n = 30), FETZ (N = 30), INSTEP (N = 4), and DUPS-U (N = 4). Similarly, subjects who were already psychotic as reported in the corresponding article were filtered out: EDIE-NL [psychotic at inclusion (N = 4), history of psychosis (N = 1)]. Subjects’ data were censored to the primary study protocol-stated follow-up period: FePsy (N = 1) and CAYR (N = 4).

Because of these procedures, a final sample of 1,676 individuals fulfilled the inclusion criteria and was included in the IPD-MA. Key details of the included studies are summarized in **Table 1**, and a more comprehensive information on each study is included in **Supplement IV**.

**Table 1:** Overview of studies utilized for the development and validation of the prognostic prediction model.

Study	Country	Inclusion period	CHR	Positive psychotic symptoms	Negative psychotic symptoms	Functioning	Transition criteria	N (% m)	Age (M, SD)	Follow-up (months)	Transition status at last follow-up (n, %)
ADAPT	Can	2008–2010	SIPS/SOPS	SIPS/SOPS	SIPS/SOPS	GAF	SIPS/SOPS	49 (73.4%)	21.3 (3,9)	24	3 (6.1%)
CAYR*	Can	2005–2014	CAARMS	BPRS	SANS	GAF	CAARMS	176 (55.7%)	19.3 (4.0)	12	16 (9.0%)
DUPS-A	NLD	2002–2006	SIPS/SOPS	SIPS/SOPS	SIPS/SOPS	GAF	PANSS	69 (66.7%)	20.0 (3.7)	36	18 (26.1%)
EDIE-NL	NLD	2008–2012	CAARMS	CAARMS	CAARMS	SOFAS	CAARMS	195 (49.2%)	22.7 (5.4)	18	32 (16.4%)
EDIE-UK	UK	1999–2002	PANSS	PANSS	PANSS	GAF	PANSS	58 (68.9%)	22.2 (4.5)	36	13 (22.4%)
FePSY*	CH	2000–2015	BSIP	BPRS	SANS	GAF	BPRS	133 (31.8%)	24.2 (5.2)	12–78	38 (28.8%)
FETZ	D	1998–2003	SIPS/SOPS	SIPS/SOPS	SIPS/SOPS	SOFAS	BPRS	161 (63.3%)	25.3 (6.1)	12–72	72 (44.7%)
GRAPE	KOR	2007–2011	SIPS/SOPS	SAPS	SANS	QLS	SCID-I	60 (58.3%)	19.7 (3.3)	20,7	14 (23.3%)
INSTEP*	JPN	2008–2013	SIPS/SOPS	PANSS	PANSS	GAF	SIPS/SOPS	53 (56.6%)	24.0 (8.4)	36	6 (11.3%)
OASIS*	UK	2013–2016	CAARMS	CAARMS	CAARMS	GAF	CAARMS	51 (58.8%)	22.8 (5.2)	17,7	16 (31.4%)
PACE	AUS	1993–2006	CAARMS	BPRS	SANS	GAF	CAARMS	415 (48.2%)	19.4 (3.4)	12–168	114 (27.7%)
PORT*	POL	2010–2016	CAARMS	CAARMS	CAARMS	SOFAS	PANSS	107 (45.8%)	18.8 (3.5)	12–84	20 (18.7%)
Rome*	ITA	2012–2013	SIPS/SOPS	PANSS	PANSS	cGAS	SIPS/SOPS	19 (52.6%)	15.3 (1.3)	12–24	5 (26.3%)
SAFE	JPN	2004–2012	CAARMS	PANSS	PANSS	GAF	CAARMS	106 (62.3%)	20.0 (4.4)	28.8	14 (13.2%)
DUPS-U	NLD	2003–2006	SIPS/SOPS	SIPS/SOPS	SIPS/SOPS	mGAF	SIPS/SOPS	25 (40.0%)	16.6 (1.6)	60	7 (28%)

AUS, Australia; BPRS, Brief Psychotic Rating Scale; BSIP, Basel Screening Instrument for Psychosis; CAARMS, Comprehensive Assessment of At Risk Mental State; CAN, Canada; cGAS, children Global Assessment Scale; CH, Switzerland; DSM-IV, Diagnostical and Statistical Manual of mental disorders version IV; GAF, Global Assessment of Functioning scale; ITA, Italy; JPN, Japan; KOR, South Korea; m, male; M, mean; mGAF, modified Global Assessment of Functioning scale; NLD, the Netherlands; PANSS, Positive and Negative Syndrome Scale’ POL, Poland; QLS, Quality of Life Scale; SANS, Scale for the Assessment of Negative Symptoms; SD, standard deviation; SIPS/SOPS, Structured Interview of Prodromal Symptoms/Scale of Prodromal Symptoms; CHR, Ultra High Risk; UK, United Kingdom.

*Data from the specified study, yet not identical to the data in the published paper, for instance, a subsample of the study or sample with a shortened or prolonged follow-up then reported in the original paper.

An overview of the comparison of study characteristics is presented in **Table 2**. The CHR-P studies worldwide participated in the study, and majority of the studies took place in Europe (53–57, 60, 62, 63, 65). Three studies concerned an RCT (51, 54, 55), one study had a mixed design of both RCT and naturalistic observational design (61), whereas all the others had a naturalistic observational design. The earlier studies started including individuals in 1993 (61), whereas the later studies started including in 2013 (60). The inclusion period varied between 1 year (52) and 13 years (61). The smallest study contained 19 subjects (63), whereas the largest study contained over 400 individuals (61). Despite methodological differences, one inclusion criterion was shared by all studies, namely, meeting the clinical high-risk criteria of at least one of the high-risk groups [genetic risk and deterioration (GRD), attenuated psychotic symptoms (APS), or brief limited psychotic symptoms (BLIPS)]. Eleven studies had additional age criteria (52, 54, 55, 58–64), one study included only participants with a minimum of 9 years of education (58); and as additional criterion for another study, individuals should have no history of antipsychotic medication for over 16 weeks (59). There was a greater variety in the applied exclusion criteria, with only the EDIE-UK (55) study that did not exclude subjects in case of a known organic cause for the presentation of prodromal symptoms. Twelve studies excluded individuals with either a current or a lifetime psychotic condition (51, 54, 58, 60–68). Ten studies excluded individuals with lower intellectual capacities (51, 52, 54, 56, 57, 59, 60, 62,, 65), five studies excluded individuals in case of substance use or abuse (52, 59, 60, 63, 64). Current or a history of antipsychotic medication was an exclusion criterion in six studies. Two studies excluded individuals with insufficient competence of the primary language (54, 66). The presence of a pervasive developmental or autism spectrum disorder was an exclusion criterion in two studies (52, 59). In one study, a history of electroshock therapy (59), withdrawing their willingness to be followed by the service (60) or suicide risk due to personality disorder (64) was an exclusion criterion. In the final database, the mean follow-up time was of 32.37 months (SD, 31.59 months), and there were 386 (23.0%) transitions to psychosis (events). Therefore, the final event per variable ratio was 1:48, which is below the threshold recommended for building robust prognostic models (14).

**Table 2:** Summary of study characteristics.

	N (studies)	% of studies	% of total sample
Continent: Europe Australia Northern America Asia	9 1 2 3	60.0 6.7 13.3 20.0	48.8 24.8 13.4 13.1
Design: Naturalistic observational RCT Mixed	11 3 1	73.3 20.0 6.7	82.0 18.1 24.8
Start inclusion period: Before 2000 2000–2005 2005–2010 2010–	3 4 5 3	20.0 26.7 33.3 20.0	37.9 11.9 31.9 10.6
Inclusion period—duration: 1 year 1–2 years 2–3 years >3 years	1 5 4 5	6.7 33.3 26.7 33.3	10.5 22.3 17.1 48.6
Information campaigns Yes No	8 7	53.3 46.7	50.0 50.0
Inclusion strategies Referral Mixed	12 3	80.0 20.0	71.4 28.6
Inclusion criteria: in additional to CHR-group: Age at inclusion A minimum of 9 years of education No history of antipsychotic medication for over 16 weeks	10 1 1	66.7 6.7 6.7	74.1 3.6 3.2
Exclusion criteria: Organic cause for prodromal symptoms Current or lifetime psychosis Intellectual functioning Substance use Current or history of antipsychotic medication Language requirements Diagnosed with pervasive developmental disorder or autism spectrum A history of electroshock therapy Not help seeking individuals Suicide risk due to personality disorder	14 12 11 5 6 2 2 1 1 1	93.3 80.0 73.3 33.3 40.0 13.3 13.3 6.7 6.7 6.7	96.7 82.9 67.1 24.2 53.8 19.5 13.7 3.0 3.0 6.3
Assessment of ultra high risk: SIPS/SOPS CAARMS PANSS BSIP	7 6 1 1	46.7 40.0 6.7 6.7	26.1 63.1 3.5 7.9
Assessment of positive psychotic symptoms: BPRS CAARMS SIPS/SOPS PANSS SAPS	3 3 5 3 1	20.0 20.0 33.3 20.0 6.7	43.2 21.1 21.1 13.9 3.6
Assessment of negative psychotic symptoms: SANS PANSS SIPS/SOPS CAARMS	4 4 4 3	26.7 26.7 26.7 30.0	46.8 13.6 18.2 21.1
Assessment of functioning: GAF SOFAS mGAF cGAS QLS	9 3 1 1 1	60.0 20.0 6.7 6.7 6.7	66.3 27.7 1.5 1.0 3.6
Transition criteria: CAARMS SIPS/SOPS PANSS BPRS SCID-I	5 4 3 2 1	46.7 26.7 13.3 13.3 6.7	56.3 8.7 14.0 17.5 3.6
Sample size: <50 50–100 100–150 150–200 >200	3 5 3 3 1	20.0 33.3 20.0 20.0 6.7	5.6 17.4 20.6 31.8 24.7
Transition rate: <10% 10–20% 20–30% 30–40% >40%	2 4 7 1 1	13.3 26.6 26.7 6.7 6.7	13.4 27.6 46.5 3.0 9.6
Treatment: CBT (RCT) Additional treatment None	3 6 6	20.0 40.0 40.0	18.1 59.0 23.1

BPRS, Brief Psychotic Rating Scale; BSIP, Basel Screening Instrument for Psychosis; CAARMS, Comprehensive Assessment of At-Risk Mental State; CBT, Cognitive Behavioral Therapy; cGAS, children Global Assessment Scale; CHR, clinical high risk; GAF, Global Assessment of Functioning scale; mGAF, modified Global Assessment of Functioning scale; PANSS, Positive and Negative Syndrome Scale; QLS, Quality of Life Scale; RCT, Randomized Controlled Trial; SANS, Scale for the Assessment of Negative Symptoms; SCID-I, Structured Clinical Interview for DSM-IV; SIPS/SOPS, Structured Interview of Prodromal Symptoms/Scale of Prodromal Symptoms.

Eight of 15 studies launched special information campaigns, either targeting only potential sources of participant referrals or the general public (51, 52, 55, 60, 62, 64, 66, 67). The campaigns differed in their elaborateness: from a website and folders to workshops, letters in newspapers, and advertisement on radio and television. All studies included individuals that were referred to them, but a few studies combined this with the option of self-referral (52, 62), referral by a close friend or family member (52) or screening in a help-seeking population (54). Six studies offered additional treatment, such as case management, cognitive behavioral therapy, psychoeducation for the CHR individuals, as well as for family, medication, sport, and nutrition groups (52, 60–62, 66, 64). Information on specific treatments that were offered was only available for RCTs, and most studies did not keep detailed records of offered interventions.

With regard to the assessment of CHR-P, symptoms, and functioning, four instruments were applied to determine whether an individual met the CHR criteria, namely, PANSS (44), CAARMS (2), the Basel Screening Instrument for Psychosis (BSIP) (4), and the SIPS/SOPS (3). Positive psychotic symptoms were assessed with five different instruments: PANSS (44), CAARMS (2), BPRS (43), SIPS (69), and the Scale of Assessment of Positive Symptoms (SAPS) (70). Negative psychotic symptoms were measured with four scales: PANSS (44), Scale of Assessment of negative symptoms (SANS) (71), CAARMS (2), and the SIPS (3). Functioning was assessed with five scales, namely, the Global Assessment of Functioning (GAF) (72), the Modified-Global Assessment of Functioning (m-GAF) (73), the Children Global Assessment Scale (cGAS) (74), the Social and Occupational Functioning Scale (SOFAS) (75), and the Quality of Life Scale (QLS) (76). Transition to psychosis was determined with four different transition criteria: CAARMS [five studies (52, 54, 60, 61, 64)], SIPS/SOPS [four studies (51, 59, 63, 65)], PANSS [three studies (53, 55, 62)], BPRS [two studies (56, 57)], and SCID-1 [one study (58)].

### Quality Assessment of Individual CHR-P Studies

All CHR-P studies received the maximum score of 4 for assessing the study quality with the NOS (40): an adequate check that outcome is not present at the start of the study, an adequate duration of the follow-up period, and an adequate proportion of participants in the follow-up assessments (see **Supplements 1** and **2**). The three RCTs additionally received an extra point for blind assessments.

### Data Cleaning and Preparation

#### Missing Data and Multiple Imputations

In the original sample, 78.6% had data on all variables. There were missing data with regard to attenuated negative psychotic symptoms (7.2%), functioning (6.6%), attenuated positive psychotic symptoms (4.8%), CHR-P group (4.2%), age (<0.1%), and sex (<0.1%). For the individuals, 3.8% were omitted from the analyses because of missing of follow-up data. There were no differences between CHR-P subjects with and without follow-up with regard to age, gender, type of CHR-P subgroup, attenuated negative psychotic symptoms, and functioning at baseline. Only the severity of attenuated positive psychotic symptoms at baseline was significantly higher for CHR-P individuals without follow-up (t = −6.244, df = 1,563, p < .001).

As noted above, the 15 included CHR-P studies had applied a variety in assessment instruments with regard to attenuated positive psychotic symptoms, attenuated negative psychotic symptoms, and functioning (see **Table 1**). All measurements were tested as the core parameters on the basis of the protocol, yet, although other instruments were applied in more individuals, attenuated negative psychotic symptoms—total score SIPS, attenuated positive psychotic symptoms—total score SIPS and GAF were selected because these had the best predictive performance. SIPS/SOPS is a frequently used instrument in the enclosed studies and is one of the golden standard measurements for positive and negative psychotic symptoms in CHR research (77). For functioning, the primary parameter is the frequently applied GAF (72). However, because the SIPS were only applied by 18.2% and the GAF by 66.3% of the individuals, there were missing data for 81.8% (attenuated positive and negative psychotic symptoms) and 33.7% (functioning). Multiple imputations were performed with 50 iteration sets. The data from the variables age, gender, GRD, APS, BLIPS, and functioning (GAF) were used to predict the missing SIPS-positive and -negative psychotic symptoms scores. The imputations diagnostics are presented in **Supplement III**.

### Testing Collinearity

An overview of the estimated VIFs is presented in **Table 3**. Overall, the majority of the predictor variables showed a VIF close to 1, indicating low shared variance with the other variables. However, the three CHR-P subgroups showed a high level of collinearity. To investigate the influence of the collinearity, all three predictors were one-by-one subsequently omitted from the analysis, leading to a drop in VIF scores of below three, yet barely influencing the outcome of the produced model. Given our aim to develop a prognostic model in which all predictors are assessed for their relative contribution to risk, these predictors were retained in further analysis, in line with the methodological recommendations (14).

**Table 3:** Predictor variables and accompanying VIF.

	Dependent								
		Gender	Age	GRD	APS	BLIPS	Pos Sx	Neg Sx	Functioning
Independent	Gender	—	1.029	1.028	1.028	1.028	1.028	1.005	1.028
	Age	1.021	—	1.022	1.022	1.020	1.012	1.016	1.022
	GRD	7.857	7.875	—	1.026	1.599	7.877	7.877	7.877
	APS	15.127	15.157	1.975	—	1.790	15.122	15.162	15.142
	BLIPS	9.848	9.848	2.004	1.165	—	9.851	9.870	9.855
	Positive Sx	1.415	1.404	1.419	1.415	1.416	—	1.208	1.418
	Negative Sx	1.833	1.867	1.879	1.879	1.879	1.599	—	1.339
	Functioning	1.586	1.589	1.590	1.588	1.587	1.589	1.133	—

APS, attenuated psychotic symptoms; BLIPS, brief limited psychotic symptoms; GRD, genetic risk and deterioration; Sx, symptoms; VIF, variance inflation factor.

### Development and Validation of the Prognostic Model

**Figure 2:**
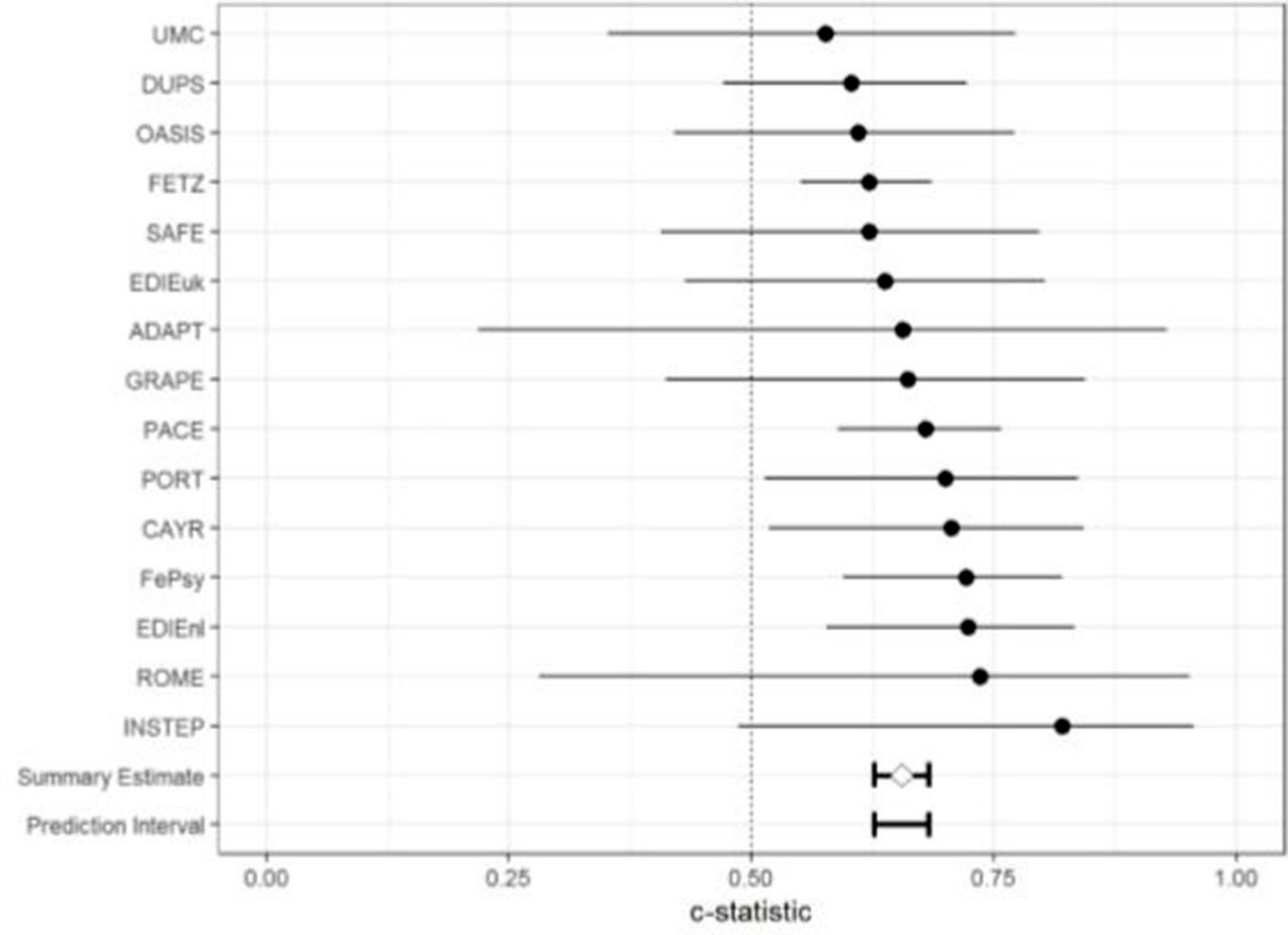
Forest plot of the discriminative ability of the model in the individual studies and its 95% CI, assessed with the C-statistics.

A parametric survival model with a log-normal distribution is fitted for event times (47): transition to psychosis from a CHR-P stage and time to transition. **Supplement V** displays the discriminative performance of the prognostic model in the individual studies at 12 and 24 months. **Figure 2** shows a forest plot with the 95% CI of the Harrell’s C-statistics of the prognostic model per study and the overall C-statistics.

The C-statistic of the model was 0.655 with a 95% CI of 0.627 to 0.682 and (approximate) 95% prediction interval of 0.614 to 0.695. Inspection of the forest plot showed that the prognostic performance in the larger studies reached an adequate level, with C-statistics of around 0.700 and 95% CI between 0.54 and 0.87 (52, 54, 56, 57, 61, 62). This is also visible in the boxplots of the individual studies (see **Supplement V**): the proportion of predicted survival per risk group is relatively equal to the observed proportion, meaning that the model can adequately discriminate between CHR-P individuals with a higher versus lower risk of developing psychosis (one survival). Yet, smaller studies have lower discriminative adequacy: in the forest plot, the 95% CIs of these studies were broad and included 0.5, which indicated that the model did not discriminate better than chance.

**Figure 3:**
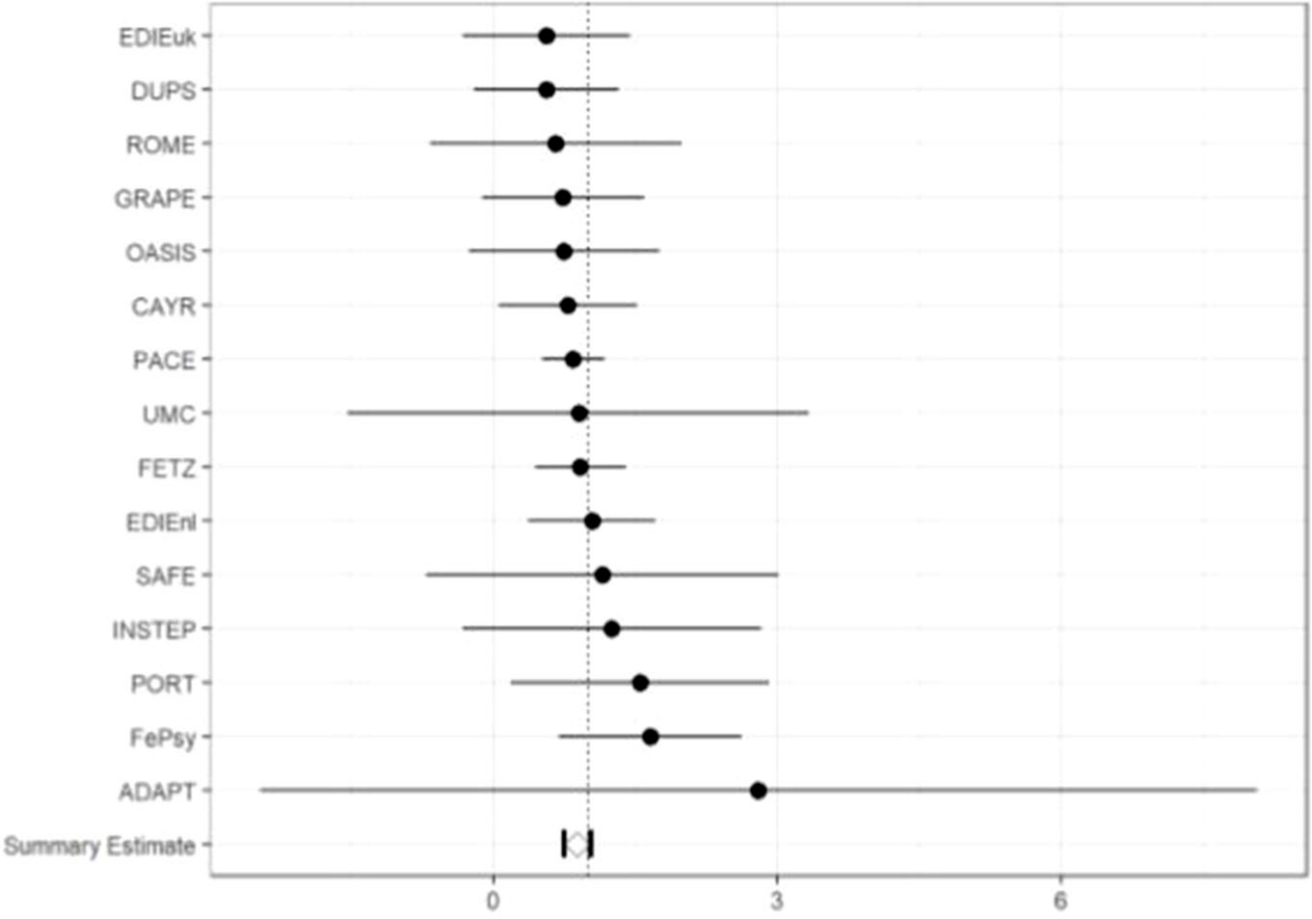
Forest plot of the external validation of calibration slope and its 95% CI in the individual studies.

The calibration slope of the model in the individual CHR-P studies, as well as overall calibration, is displayed in **Figure 3**.

The internal–external validation results for the calibration slope gives an overall estimate of 0.886 (95% CI, 0.745–1.022), which indicated that at 2 years, the predicted probabilities, on average, vary too much. Because the 95% CI includes 1, the overall calibration slope yields as non-significant. Calibration slopes of the individual studies not overlapping with 1 indicate no need for recalibration. Inspection of the forest plot showed that all studies overlapped with 1, which indicated that the prognostic model calibrates sufficiently well, and there are no direct indications that the parameters of the model should be adjusted with shrinkage methods.

#### Final Model

**Table 4** presents the final model with its intercepts; all included predictors have a significant contribution to the prediction, as tested with an independent sample *t* test. The scale parameter is 2.119.

**Table 4:** Variables and intercepts of the final model.

Variable	Intercept:	T	SE of Mean	Sign.	95% confidence interval	
					Lower	Upper
Intercept	7.543328648	51.792	.14565	<.001	7.251	7.836
Sex—female	0.179071582	13.247	.01352	<.001	0.152	0.206
Age	−0.048979637	−42.162	.00116	<.001	−0.051	−0.047
APS—yes	−0.369616434	−7.737	.04777	<.001	−0.466	−0.274
BLIPS—yes	−0.738429338	−15.950	.04630	<.001	−0.831	−0.645
Functioning: GAF score	0.006634737	4.059	.00163	<.001	0.003	0.010
Negative psychotic symptoms: SIPS/SOPS—total score	−0.054490819	−14.542	.00375	<.001	−0.062	−0.047
Positive psychotic symptoms: SIPS/SOPS—total score	−0.092850985	−16.356	.00574	<.001	−0.105	−0.082

APS, attenuated psychotic symptoms; BLIPS, Brief Limited Psychotic Symptoms; GRD, Genetic Risk and Deterioration; SE, Standard Error; Sign, significance level; SIPS/SOPS, Structured Interview of Prodromal Symptoms/Scale of Prodromal Symptoms.

### Prognostic Prediction for Individual CHR-P Patients

With a parametric survival model with a log-normal distribution for event times, a (cumulative) survival probability can be calculated for time (*t*) in CHR-P individual subjects, utilizing the linear predictor (5.777) and the earlier reported scale parameter (78).

The following formula that estimates the risk of psychosis (1 survival) for an individual patient derives from the model:

Risk of psychosis = 1 − (7.543 + 0.179 (sex = female) + −0.049 × (age)+.689 × (genetic risk and deterioration) + −0.370 × (attenuated psychotic symptoms = yes) + −0.738 × (brief limited intermittent psychotic symptoms = yes) + 0.006 × (functioning GAF) + −0.052 × (total score negative psychotic symptoms SIPS/SOPS) + −0.102 × (total score positive psychotic symptoms SIPS/SOPS)).

### Case Study

Considering a 21-year-old female that meets the CHR-P criteria of brief intermittent psychotic symptoms, with baseline GAF score of 65, SIPS/SOPS attenuated negative psychotic symptoms total score of 13 and a SIPS/SOPS attenuated positive psychotic symptoms total score of 8, the predicted 2-year survival would be 0.835. This implies that her probability of developing psychosis within the first 2 years is 1 −.835 = .165, which is of about 16%.

### Heterogeneity

The 95% prediction interval of the C-statistics (0.614–0.695) shows a moderate range, which indicates that there is substantial heterogeneity between the predictions of the model in the different studies. There is a larger amount of heterogeneity detectable with regard to the overall calibration slope which shows a rather large 95% CI of 0.745–1.022. This is supported by the large variety in operationalization of symptoms in the different assessment instruments, as well as variety in outcome criteria.

## Discussion

The aim of this study was to develop and validate a prognostic model based on clinical predictors that are available in clinical routine for forecasting the onset of a psychotic episode in CHR-P individuals, using an IPD-MA. The predictors were selected *a priori* as recommended by state-of-the-art prognosis guidelines. The predictors encompassed two demographical predictors (age, gender) and six clinical predictors collected at baseline (genetic risk and deterioration syndrome CHR-P subgroup, attenuated psychotic symptoms CHR-P subgroup, brief and limited intermittent psychotic symptoms CHR-P subgroup, severity of attenuated positive psychotic symptoms, severity of attenuated negative psychotic symptoms, level of functioning) predictors. The overall model achieved a C-index of .655, indicating a modest subject-level ability to differentiate between CHR-P individuals with a high-risk likelihood that develop psychosis from those at lower risk. The overall calibration slope indicated that the model can significantly distinguish CHR-P individuals who convert to psychosis versus those who do not. Most of the included predictors showed a significant contribution to the model, with the exception of CHR-P group membership (which was characterized by high collinearity). The removal of these variables from the model indicated that the influence of this collinearity on the final model was non-significant and minor in magnitude.

This is the first IPD-MA and the largest clinical prediction modeling study conducted in the CHR-P field. Indeed, one of the main advantages of developing a prognostic model using an IPD-MA is the possibility of reaching large sample sizes, which enables the building of a more robust prediction model. Moreover, the model’s generalizability can be strengthened by the inclusion of several large data sets from all over the world. Ensuring appropriate representativeness of CHR-P samples is pivotal to developing robust prognostic models because of the severe sampling biases that affect this population (18, 79, 80). Our approach was partially successful. On one side we demonstrated that our *a priori* selected predictors did produce a prognostic model that forecasted the onset of psychosis at the individual subject level with an accuracy superior to chance (0.655). From a methodological point of view this confirms that preselecting predictors on the basis of previous knowledge and using all of them in the prognostic model is a robust way for developing risk prediction algorithms. On the other side, the level of accuracy was only moderate. This could be due to the fact that our IPD-MA combined CHR-P studies employing different definitions of predictors and outcomes, and that there were some missing data (81). Furthermore, to ensure a prognostic model that could easily be applied in clinical practice, we decided to use only one instrument per predictor (e.g., the SIPS and not the CAARMS, PANSS, SAPS, or BPRS, and the GAF and not the SOFAS, mGAF, cGAS, or QLS). This was prespecified at the PROSPERO protocol level. This decision resulted in missing data, which has to be considered as missing not at random (MNAR). The problem was particularly severe because this led to a rather high level of missing data (81.8% for the attenuated positive/negative psychotic symptoms and 33.7% for the level of functioning). Although the missing data were handled with the recommended multiple imputation techniques (82), it did imbalance the final prognostic model. These choices counterweight the moderate prognostic accuracy of our model because they facilitate its theoretical implementability in clinical routine. Scalability of prognostic models is an essential criterion that should be fully considered beyond the level of prognostic accuracy. In fact, even prognostic models that have a suboptimal (but clearly higher than random prediction) level of prognostic performance can be clinically useful if they can enter clinical routine at scale. For example, a prediction model has recently been developed and validated using a patient data and machine learning to predict treatment outcome in depression: the overall performance of this model was of a very similar moderate prognostic performance (0.65) (83).

The next stage would be to refine and improve this model. The first option would be to consider using advanced machine-learning approaches. Yet, there is no strong evidence that these methods can deliver more robust and implementable prognostic models compared with *a priori*–defined statistical models. Interestingly, although the prognostic model described above leveraged machine learning methods, its overall prognostic performance was of a similar level than that of our current model (83). A recent systematic review conducted by methodologists showed no performance benefit of machine learning over logistic regression for clinical prediction models (84). However, it is possible that machine learning methods could demonstrate some clear advantages with the addition of multidimensional predictors encompassing neurobiological, genetic, and other modalities (14). The downside of multimodal approaches is that they tend to deliver more complex prognostic models at the expense of scalable implementability. This IPD-MA study also calls for more homogeneity in the CHR-P assessment instruments or at least more research in the development of converting formulas. This would have allowed minimizing missing and imputed data. For example, a between-assessment scale converter algorithm for symptom rating in schizophrenia has been developed by van Erp et al. (85), which enabled both researchers as clinicians to convert the scores of positive and negative psychotic symptoms assessed by the PANSS, SANS, and SAPS. Similarly, an automatic Phyton package called “convert” has been developed to convert CAARMS into SIPS scores and vice versa (36). The tool is freely available online at https://bitbucket.org/ioppn/convert. Unfortunately, we did not have the raw data on the specific CAARMS or SIPS (P1–P5) subscales to use this package, but we only had the overall severity of attenuated positive/negative psychotic symptoms across these two instruments. Beyond the diversity in the assessment instruments, there is another cause of suboptimal prognostic performance for our model, which is the baseline intrinsic difference in study populations. This is supported by the finding that there is substantial diversity in baseline risks and by the finding that our prognostic model had an adequate level of performance (C-statistic 0.7) in the subset of the largest CHR-P studies. These studies are likely to be those with the highest-risk enrichment and less affected by sampling biases which are particularly serious in the case of small CHR-P studies. A meta-analysis by Fusar-Poli et al. (86) demonstrated that these sampling biases are mostly due to the way CHR-P individuals are being recruited for undergoing the initial assessment. Specifically, recruiting CHR-P individuals mostly from the community would dilute the risk enrichment (and therefore the transition risk) compared with samples mostly recruited through the secondary mental health care system. This was also reflected by the type of outreach campaigns adopted by each CHR-P clinic. In comparison to CHR-P studies that targeted their outreach campaigns to health care referrers, CHR-P studies with outreach campaigns that were focused on the general public were associated with lower risk of psychosis. There was also a clear relation between the intensity of the campaign (amount of activities) and a diminished transition risk. In our IPD-MA, CHR-P studies differed strongly with regard to information campaigns, as well as sources of referrals, and this factor may have amplified sampling biases and reduced the prognostic performance of our model.

Another factor that could have modulated the prognostic accuracy of our model may have been the preventive treatments offered to the CHR-P individuals. An earlier meta-analysis (87) examined the preventive effects of antipsychotic medication, dietary supplements, integrated psychological treatments, and cognitive behavioral therapy on the transition to psychosis and reported an overall risk reduction pooled across all of these categories of 54% at 12 months and of 37% at 24 months. However, the evidence remains inconclusive while a more recent network meta-analysis which included about 1,000 more CHR-P individuals found no evidence to favor specific preventive treatments compared with each other for the prevention of psychosis (88).

## Limitations

One limitation of the current study is that it did not account for treatment effects. The majority of the included studies have a naturalistic, observational design, and as such are an adequate reflection of current clinical practice. Since subject-level data on preventive interventions were only available for RCTs (51, 54, 55), the effects of these treatments could not be entered into the model, and as such their effects could not be controlled for. However, as indicated above, the actual effectiveness of preventive treatments for CHR-P individuals is questionable. As such, it is unlikely that this factor would have impacted our findings substantially. Another limitation is that documented clinical predictors in transition risk could not be used in our model because these were not recorded in the majority of the studies. These predictors are for instance childhood adversities, cognitive biases, social cognition, verbal fluency, beliefs of social marginalization, subjective complaints about motor functioning, urbanicity, and poor premorbid social adjustment. The prediction model could be improved if future studies into risk assessment would measure these risk factors systematically. The main limitation of this IPD-MA was that we were only able to collect a minority of the available data. Because of the sampling biases discussed above, this represents a major barrier to generalizability. It is clear that future IPD-MAs in CHR-P populations face the difficult challenge of collecting all (at least 80%) of the potential studies identified. The additional limitation is that we had to disregard some data because of the high heterogeneity of the measurements. Future IPD-MA could benefit from the converting strategies across different scales that have been discussed above here.

## Clinical Implications

Given the above caveats, implementing the current prediction model in clinical practice is not desirable. This does not imply that the model is overall redundant. Future refinement of the model in specific clinical circumstance can be considered. For example, future research can clarify the characteristics of the largest studies in which this model can perform better. An answer to this question is rather complex, since these studies vary greatly with regard to inclusion strategies, with studies accepting self-referrals or referrals by friend or family (52), studies that screened in help seeking populations (54), as well as specialized secondary care (57). The offered treatments varied from none (56) to studies with different treatment options (52, 61). Moreover, CAYR (52) shared data of a relatively short follow-up period of only 1 year and a transition rate of 9.0%, whereas FETZ (57) monitored their participants for up to 6 years and reported a transition rate of 44.7%.

## Further Research Directions

One avenue for further research could be to investigate whether the prognostic quality of the current model can be optimized: even though a common reaction is to develop a new prediction model, the recommendation is to iteratively adjust the model by adding new data (89). The main reason for updating the available model is the opportunity for further improving the stability and generalizability of the model by considering additional predictors. Improving the stability of the current model would result in predicted outcomes less influenced by variation in input and enhance reliability. This updating can vary between simple recalibration (adjusting the intercept of the model) and an overall adjustment of the associations of the predictors with the outcome. The most obvious option for improvement could be found in the inclusion of data from research projects identified in the systematic search that have not shared their data so far. Yet, another possibility is that IPD-MA in CHR-P could never deliver robust prognostic models, because of the inherited heterogeneity of the underlying population, assessment measurements, and preventive treatments. Such a hypothesis may suggest that future prognostic research in the CHR-P field should rather focus on conducting new large-scale prospective cohort studies that are well characterized phenotypically.

## Conclusion

This is the first IPD-MA in CHR-P individuals and the largest clinical prediction study ever conducted in these patients to date. There were 1,676 CHR-P individuals that have been used to develop and validate an individualized prognostic model based on clinical variables to forecast transition to psychosis. The model has a moderate to adequate prognostic accuracy, but there are potential options to improve its performance. At the same time, it is important to acknowledge that prognostic models based on IPD-MA may not be particularly effective in the CHR-P field. Harmonization in the CHR-P assessment instruments is a necessary step toward more homogenous databases that can support the development and validation of more robust prognostic models.

## Contribution to the Field

A psychotic disorder emerges mostly in adolescence and early adulthood and affects up to 4 in 100 individuals. The Clinical High Risk state for Psychosis (CHR-P) has become the cornerstone of modern preventive psychiatry. More recently, individualized prognostic models have been used to predict a transition to psychosis, but are typically not easily applicable in clinical practice, because required information to make a prediction requires specific equipment or training and is expensive.

In this study, we aimed to build a model to predict who will develop a psychosis based on information that is routinely collected in the clinical field. For the first time, data from CHR-P cohort studies worldwide were used to build this model. In this study we show that our model can moderately predict whether an individual develops psychosis. Despite our positive results, it is also important to acknowledge some relevant limitations. Because of the large variety between the CHR-P studies prediction models based on IPD-MAs in this population may not be able to reach higher-performance measures. Harmonization of CHR-P assessments and therapeutic interventions may be the first step to facilitate future IPD-MAs in this field.

## Data Availability Statement

The data sets for this manuscript are not publicly available because individual patient data were provided by several research groups and are official property of the researchers who conducted the original cohort and intervention studies. They shared their data solely for the purpose of this study. Requests to access the datasets should be directed to Data requests should be directed to the individual researchers of the participating studies.

## Author Contributions

The conception or design of the work was done by AM, NB, PF-P, and GP. Original study data were collected by JA, MP, DN, LH, AM, AR-R, ES, SR, FS-L, SA, SK, KK, BN, PM, SW, AL, AY, MK-A, MA, SV, MK, KM, SD, TZ, HI, MG, and PF-P. AM coordinated the data collection of the IPD-MA. Data analysis was done by Thomas Debray, whereby interpretation was performed by AM, HB, NB, PF-P, and GP. The drafting of the article was done by AM, HB, NB, and GP, and critical revisions were made by NB, SJ, Thomas Debray, PF-P, and GP. Final approval of the version to be published was given by AM, NB, HB, SJ, AA, JA, MP, DN, LH, AM, AR-R, ES, FS-L, SR, SA, SK, KK, BN, PM, AL, SW, AY, MK-A, MA, SV, MK, KM, SD, TZ, LW, HI, MG, FP-F, and GP.

## Funding

For the open access publication fees, there is funding received from the University of Groningen, NHL Stenden University of Applied Sciences, GGZ Friesland Mental Health Institute, and GGZ Drenthe Mental Health Institute, that will equally share the costs. ADAPT: JA received funding from NIMH and Alberta Heritage Foundation for Medical Research. CAYR: Research at CAYR was supported by a NARSAD Young Investigator Award to MP. DUPS: This project was supported by a research grant from The Netherlands Organization for Health Research and Development (ZonMw, 2630.0001). EDIE-NL: MG received funding from Netherlands Health Research Council, ZonMW (120510001). EDIE-UK: This research was supported by research grants from the North West National Health Service R&D Executive and the Stanley Medical Research Institute. FEPSY: This project was supported by the Swiss National Science Foundation no. 3200-057216.99, no. 3200-057216.99, and no. PBBSB-106936, the Nora van Meeuwen-Haefliger Stiftung, Basel (CH). FETZ: Data analyses were supported by a grant from the Koeln Fortune Program/ Faculty of Medicine, University of Cologne (projects 8/2005 and 27/2006); the Awareness Program was supported from 2000 to 2005 by a grant from the German Federal Ministry for Education and Research, BMBF (grant 01 GI 0235). GRAPE: This work was supported by a grant of the Korea Healthcare Technology R&D Project, Ministry for Health, Welfare and Family Affairs, Republic of Korea (A090096) and by a National Research Foundation of Korea (NRF) grant funded by the Ministry of Science, ICT & Future Planning, Republic of Korea (No. 2010-0026833, No. 2017R1A2B3008214). INSTEP: This study was supported in part by JSPS KAKENHI Grant Number JP16H06395, 16H0639X, 16K21720 & 17H05921, AMED under Grant Number JP18dm0307001 & JP18dm0307004, UTokyo Center for Integrative Science of Human Behavior (CiSHuB), and the International Research Center for Neurointelligence (WPIIRCN) at The University of Tokyo Institutes for Advanced Study (UTIAS). OASIS: PF-P was supported by the King’s College London Confidence in Concept award from the Medical Research Council (MRC) (MC_PC_16048). This study also represents independent research partially funded by the National Institute for Health Research (NIHR) Biomedical Research Centre at South London and Maudsley NHS Foundation Trust and King’s College London. PACE: funding support of National Health and Medical Research Council (NHMRC) Program grants 350241 and 566529 and the Colonial Foundation. BN was supported by an NHMRC Senior Research Fellowship (1137687), SW was supported by an NHMRC Clinical Career Developmental Award (359223), and AY was supported by an NHMRC Senior Research Fellowship (566593). PORT: Research activities regarding ARMS individuals included in the PORT program are financed by the Polish Science National Centre, grant no. NN402 1793 34. ROME: MA was supported by the Brain and Behavior Research Foundation (21278) (formerly NARSAD). SAFE: the Ministry of Education, Culture, Sports, Science and Technology (MEXT) Grants-in-Aid for Scientific Research (KAKENHI) Grant Numbers 17790803, 19591336, 22390219, and 25461747, Japan. DUPS-U: None. The funders had no influence on the design, collection, analysis, and interpretation of the data, writing of the report, and decision to submit this article for publication.

## Disclaimer

The views expressed are those of the author(s) and not necessarily those of the NHS, the NIHR, or the Department of Health and Social Care.

## Conflict of Interest Statement

PF-P received research funding and advisory board fees from Lundbeck LTD outside and not in relation to the current study. The remaining authors declare that the research was conducted in the absence of any commercial or financial relationships that could be construed as a potential conflict of interest.
