# Supplementary material for: Individualized Prediction of Transition to Psychosis in 1,676 Individuals at Clinical High Risk: Development and Validation of a Multivariable Prediction Model Based on Individual Patient Data Meta-Analysis
Source: Front Psychiatry. 2019 May 21;10:345. doi: 10.3389/fpsyt.2019.00345 (PMC6537857; doi:10.3389/fpsyt.2019.00345)

SUPPLEMENT I: NEWCASTLE - OTTAWA QUALITY ASSESSMENT SCALE (NOS) COHORT STUDIES

<u>Note</u>: A study can be awarded a maximum of one star for each numbered item within the Selection and Outcome categories. A maximum of two stars can be given for Comparability

Selection

1) <u>Representativeness of the exposed cohort</u>
a) truly representative of the average _______________ (describe) in the community ☐
b) somewhat representative of the average ______________ in the community ☐
c) selected group of users eg nurses, volunteers
d) no description of the derivation of the cohort

2) <u>Selection of the non-exposed cohort</u>
a) drawn from the same community as the exposed cohort ☐
b) drawn from a different source
c) no description of the derivation of the non-exposed cohort

3) <u>Ascertainment of exposure</u>
a) secure record (eg surgical records) ☐
b) structured interview ☐
c) written self-report
d) no description

4) <u>Demonstration that outcome of interest was not present at start of study</u>
a) yes ☐
b) no

Comparability

1) <u>Comparability of cohorts on the basis of the design or analysis</u>
a) study controls for _____________ (select the most important factor) ☐
b) study controls for any additional factor ☐ (This criteria could be modified to indicate specific control for a second important factor.)

Outcome

1) <u>Assessment of outcome</u>
a) independent blind assessment ☐
b) record linkage ☐
c) self report
d) no description

2) <u>Was follow-up long enough for outcomes to occur</u>
a) yes (select an adequate follow up period for outcome of interest) ☐
b) no

3) <u>Adequacy of follow up of cohorts</u>
a) complete follow up - all subjects accounted for ☐
b) subjects lost to follow up unlikely to introduce bias - small number lost - > ____ % (select an adequate %) follow up, or description provided of those lost) ☐
c) follow up rate < ____% (select an adequate %) and no description of those lost
d) no statement

SUPPLEMENT II. Results quality assessment based on the Newcastle Ottawa Scale (NOS)

Study	Author	Selection				Comparability		Outcome			Total
		Representativeness	Selection	Ascertainment	Demonstration	Study controls for an important factor	Study controls for any additional factor	Assessment	Duration of follow- [A]	Adequacy [B]	
ADAPT	Addington	*	Na	Na	*	Na	Na	*	*	*	5
CAYR	Pruessner	*	Na	Na	*	Na	Na		*	* [I]	4
DUPS-A	Nieman	*	Na	Na	*	Na	Na		*	*	4
EDIE-UK	Morrison	*	Na	Na	*	Na	Na	*	*	*	5
EDIE-NL	Gaag, van der	*	Na	Na	*	Na	Na	*	*	*	5
FePSY	Spitz	*	Na	Na	*	Na	Na		*	* [I]	4
FETZ	Schultze-lutter	*	Na	Na	*	Na	Na		*	*	4
GRAPE	An	*	Na	Na	*	Na	Na		*	*	4
IN-STEP	Koike	*	Na	Na	*	Na	Na		*	* [I]	4
OASIS	Fusar Poli	*	Na	Na	*	Na	Na		*	* [I, II]	4
PACE	Nelson	*	Na	Na	*	Na	Na		*	*	4
PORT	Kotlicka-Antczak	*	Na	Na	*	Na	Na		* [I]	*	4
Rome	Armando	*	Na	Na	*	Na	Na		*	*	4
SAFE	Katsura	*	Na	Na	*	Na	Na		*	*	4
DUPS-U	Ziermans	*	Na	Na	*	Na	Na		*	*	4

[A] Adequate follow-up period is set at 12 months

[B] Adequacy of follow-up cohort: minimum follow-up rate of 50-80% in cohort studies and 80% in RCTs (Kristman, 2008).

[I] Calculated from IPD-MA data

[II] Subset of a larger dataset

SUPPLEMENT III - Multiple imputations

Missing data is imputed according to Multiple Imputations with Chained Equations (MICE) with 50 iterations sets. As recommended by White and Royston (2009), the event indicator and Nelson-Aalen estimator of cumulative baseline hazard were included in the imputation model.

Imputation diagnostics

The plausibility of imputations can be assessed by studying the discrepancy between the observed and imputed data. The idea is that good imputations have a distribution similar to the observed data.

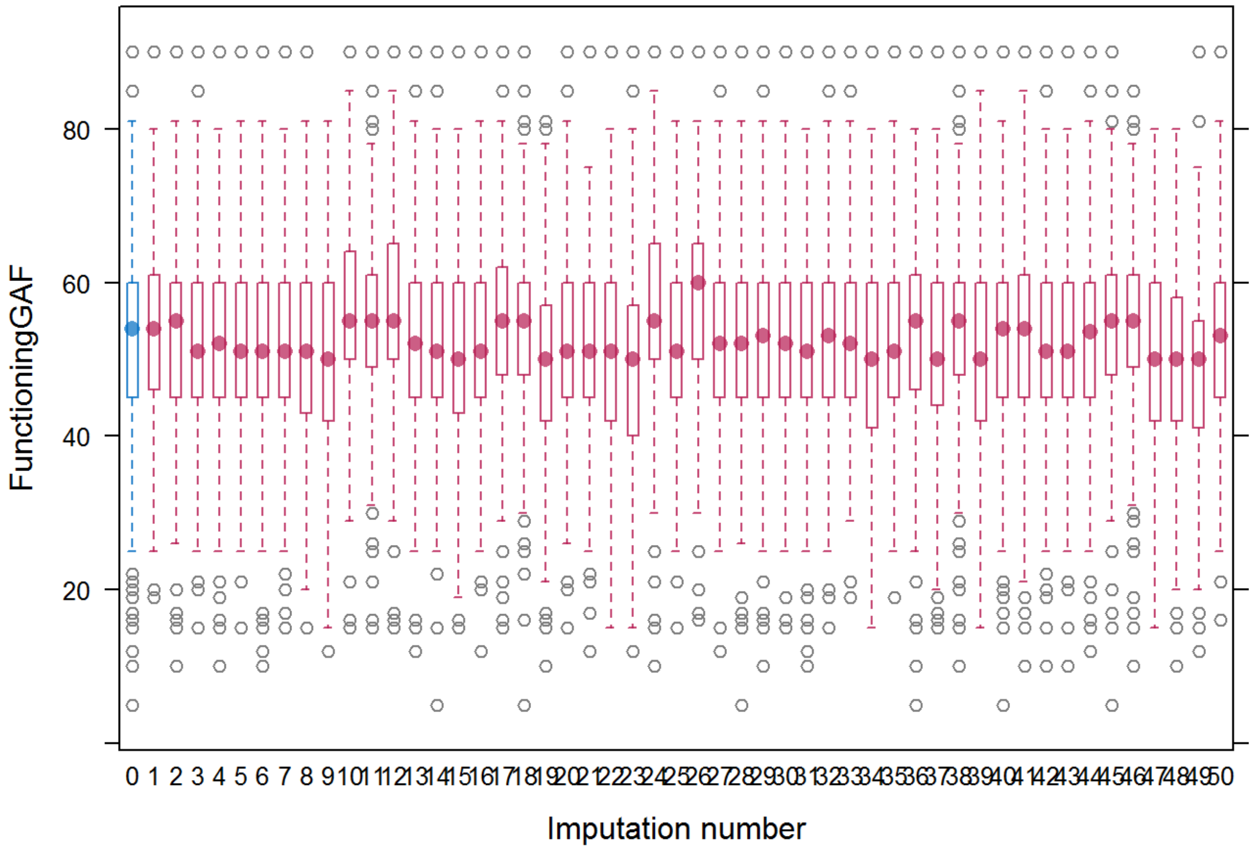

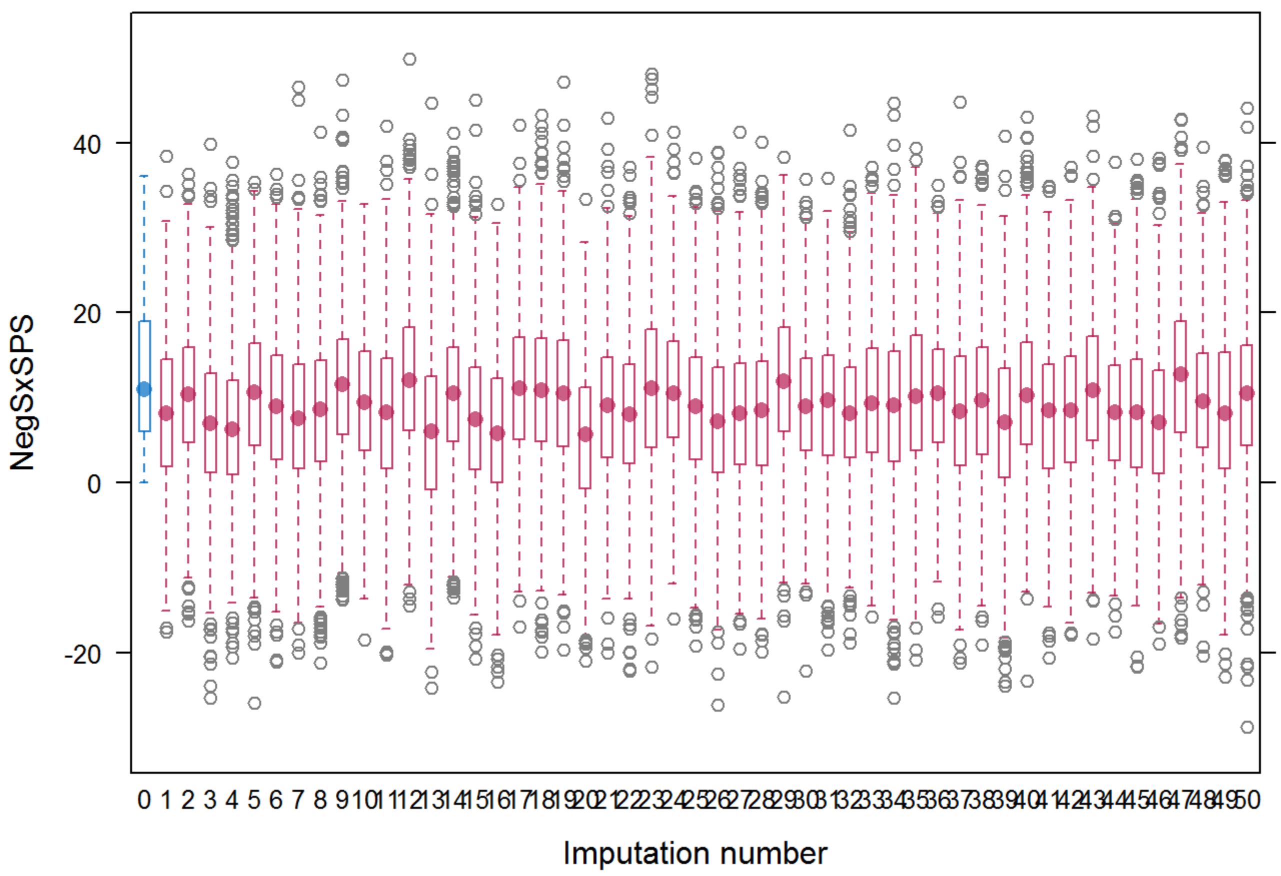

NegSxSPS
Imputation number

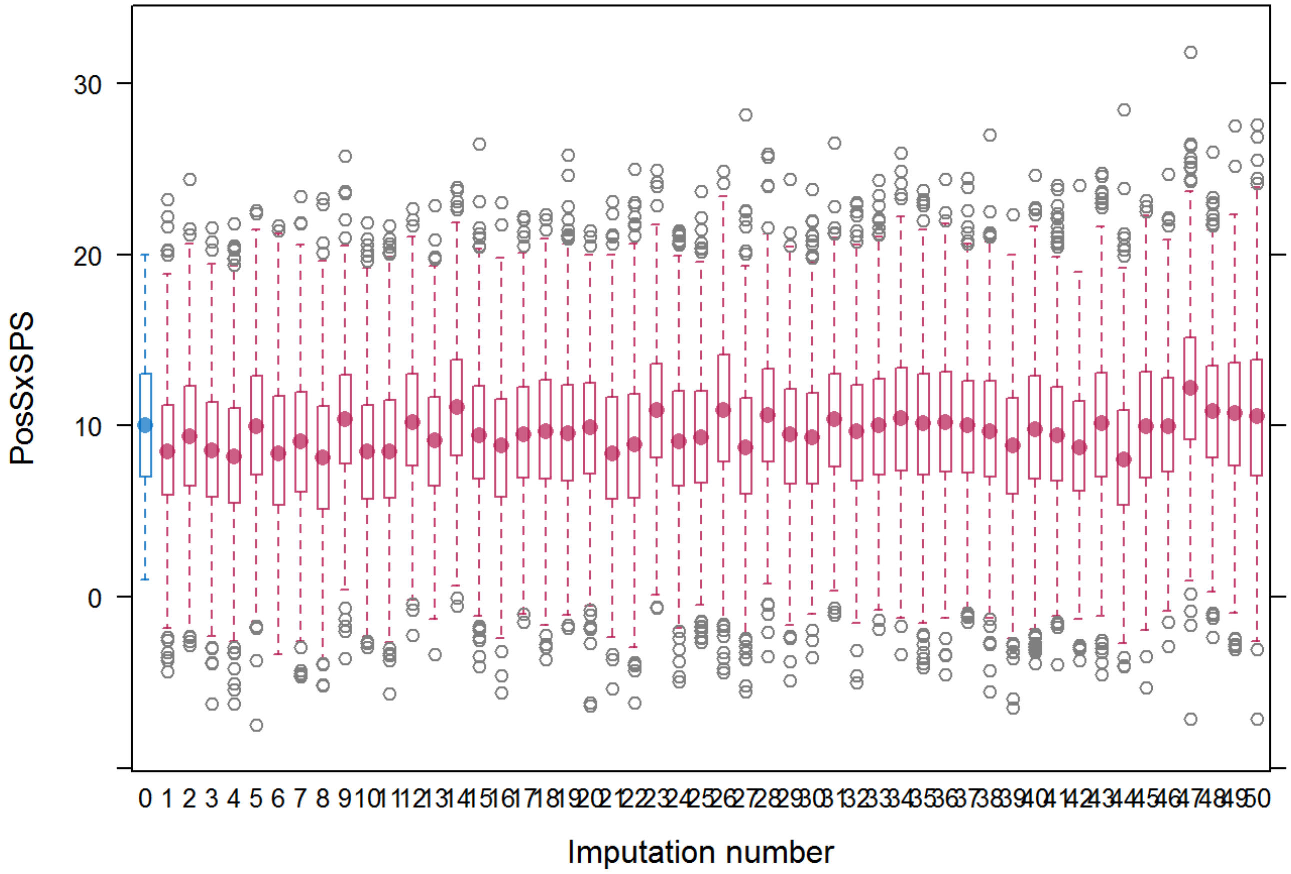

For continuous variables with many missing values we can also compare the density of original and imputed data:

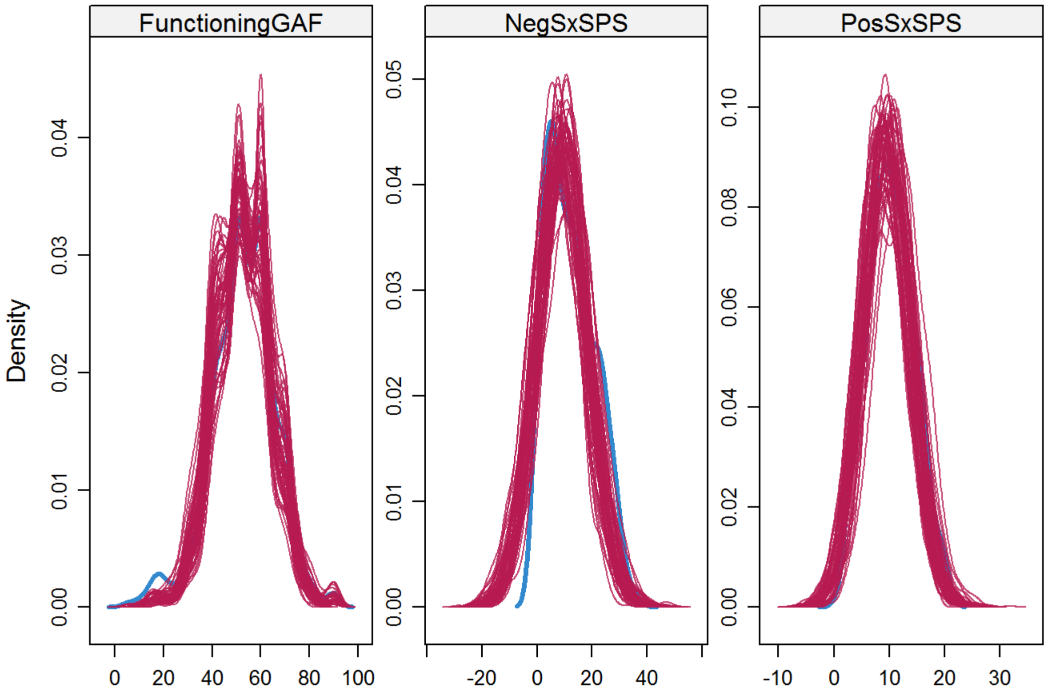

Finally, we can construct trace line plots to assess convergence of the imputation models. These plots portray the mean and standard deviation of the imputed (not observed) values against the iteration number for each of the m replications. Again, we will only do this for variables with many missings.

Functioning:

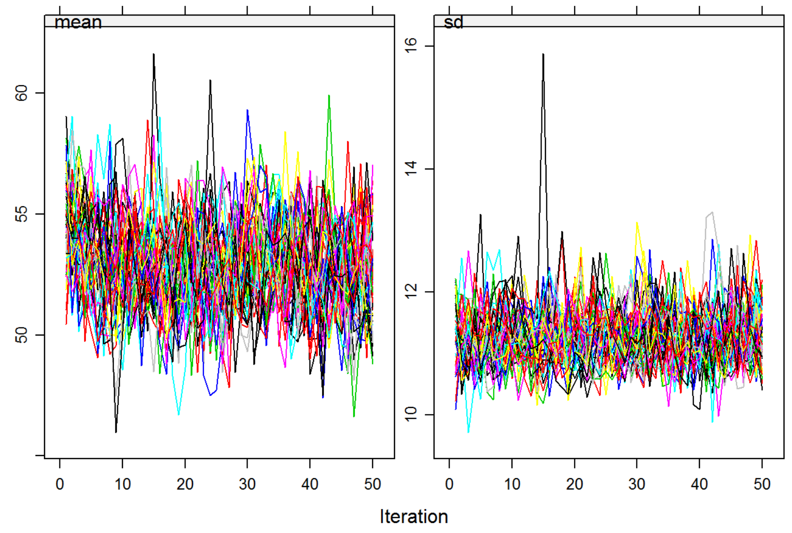

:

Negative symptoms

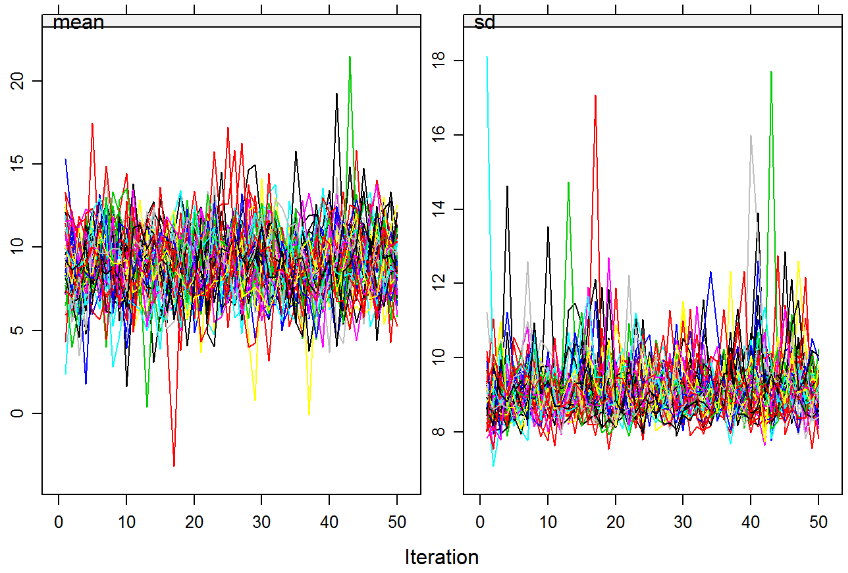

Positive symptoms:

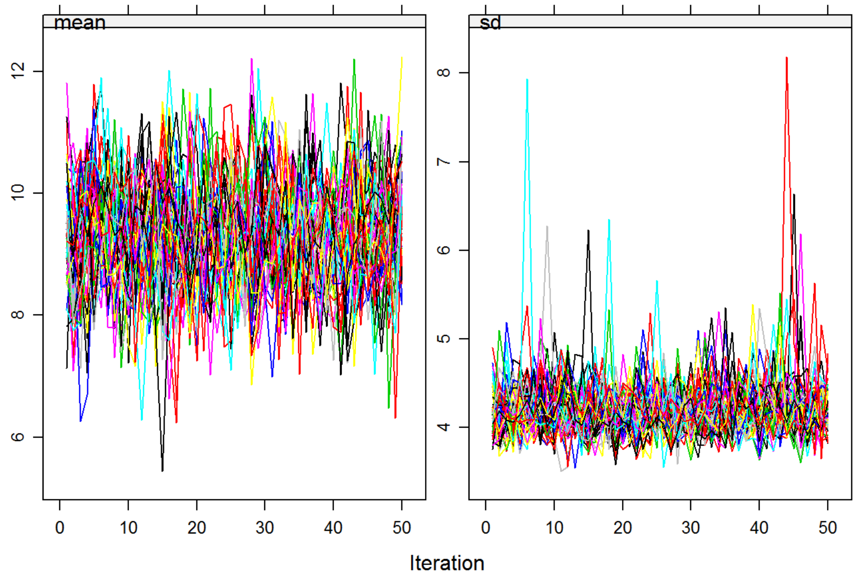

SUPPLEMENT IV: Overview of the included studies

Study	Design	Inclusion	Intervention	Inclusion criteria	Exclusion criteria	Transition criteria	Extra	Treatment
ADAPT	RCT	Referals	CBT vs Supportive therapy	1.Meeting the CHR criteria (APS, BLIPS, GRD)	1. Any current or lifetime axis I psychotic disorder, 2. prior history of treatment with an antipsychotic, 3. IQ<70 or 4. past or current history of a clinically significant central nervous system disorder which may confound or contribute to prodromal symptoms.	SIPS/SOPS	Recruitment and ascertainment methods included advertisement on radio, public transit and local newspaper	Na
CAYR	Naturalistic follow-up	Referals and self-referals or by family members	na	1.Age between 14 and 35 years 2.Presence of at least one of the CHR criteria (APS, BLIPS, GRD)	Any history of 1. Organic brain damage, 2. Pervasive developmental disorder 3. Mental retardation, 4. Epilepsy, 5.Head trauma resulting in loss of consciousness, 6. Severe substance abuse	CAARMS	Brochure, website, letters to potential referral sites, presentation offered to community clinics and hospitals.	Medication except for antipsychotica, CBT, CBT-Sad, nutrition group, family psycho-education, individual psycho-education, casemanagement
DUPS-A	Naturalistic observational follow-up	Referrals	na	1. Presence of at least one of the CHR criteria (APS, BLIPS, GRD, 2. Basic symptoms	1. a low estimated verbal IQ (IQ < 85) as assessed by the Dutch National Adult Reading test, 2. past or present psychotic episode lasting longer than 1 week (ie, fulfilling Diagnostic and Statistical Manual of Mental Disorders, Fourth Edition [DSM-IV] 3. criteria of a brief psychotic episode for at least 7 days, assessed by the Structured Clinical Interview for DSM-IV, and 4. symptoms relevant for inclusion arising from a known general medical disorder or drugs or alcohol dependency as defined by the Comprehensive International Diagnostic Interview (CIDI39). Prior use of AP no exclusion ivm naturalistic observational design.	PANSS	na	na
EDIE-NL	RCT	Screening in help seeking population and referral	CBT vs TAU	1. 14-35 years old, 2. Genetic risk or CAARMS scores in the range of ARMS, 3. Impairment in functioning (SOFAS score of ≤ 50 or drop in functioning of 30% in past year for at least a month)	1. Current or history of use of antipsychotic medication (>15 haloperidol equivalents), 2. severe learning impairment, 3. Problems due to organic condition, 4. insufficient competence of the dutch language, 5. history of psychosis	CAARMS	na	Na

EDIE-UK	RCT	Referral	CBT vs TAU	1.Meeting the CHR criteria of APS, BLIPS or GRD 2. 16-36 years old	1. current or past receipt of antipsychotic medication	PANSS	Workshops were held for all of these organisations, and regular written reminders were provided.	Na
FePSY	Open, prospective study	Referral	na	1. Meeting the CHR criteria (APS, BLIPS, GRD) according to BSIP and BPRS, 2. Unspecified risk	1. Age below 18 years, 2. insufficient knowledge of German, 3. IQ < 70, 4. previous episode of schizophrenic psychosis (treated with major tranquillisers for >3 weeks), 5. psychosis clearly due to organic reasons or substance abuse, or psychotic symptomatology within a clearly diagnosed affective psychosis or borderline personality disorder.	BPRS	Regular information campaigns with scientific symposia and teaching courses for general practitioners, psychiatrists, social service staff, etc. In addition, a public campaign with articles published in local newspapers and a special website	Medication (antidepresiva), supportive counseling, clinical management
FETZ	Naturalistic follow-up	Referal	na	1. meeting the CHR-criteria (APS, BLIPS, GRD), 2. Basic symptoms	I. Current or past diagnosis of any psychotic disorder according to DSM-IV criteria, (II) diagnosis of delirium, dementia, amnestic or other cognitive neurological disorders, mental retardation according to DSM-IV, psychiatric disorders due to a somatic factor or related to psychotropic substances according to DSM-IV and (III) general medical conditions affecting the central nervous system.	BPRS	A broad awareness program was launched, primarily aimed at mental health professionals as well as institutions and persons who might be contacted by help-seeking high risk persons. Knowledge about early warning signs and PIPS symptoms was disseminated, e.g., in local workshops, articles in professional journals and newsletters. On a smaller scale, the general public was targeted mainly by press releases to local and non-scientific papers as well as radio and television interviews.	na
GRAPE	Naturalistic follow-up	Referral	na	1. All participants met the inclusion criteria of CHR (APS, BLIPS, GRD), 2. being between 15–35 years old and 3. having more than 9 years of education.	1. current or past neurological illness or traumatic brain injury, 2. current or past major psychiatric disorder with psychotic features	SCID-I	na	na

IN-STEP	Prospective observational cohort study	referal	na	1. meeting the CHR critaria and 15-40 yrs, 2. no history of antipsychotic medication for psychosis for more than 16 cumulative weeks, 3. continious psychotic symtpoms withing the past 60 months	1. neurological illnesses, 2. previous traumatic brain injury, 3. history of ECT therapy, 4. low premorbid IQ (<70), 5. previous alcohol addiction, 6. previous continous substance use, 7. clearly diagnosted with autistic disorder	SIPS/SOPS	na	na
OASIS	Naturalistic follow-up	referral	na	1. 14-35 year old, 2. Meeting the CHR criteria (APS, BLIPS, GRD), 3. Meeting the Basic Symtpoms criteria	1. history of frank psychotic episodes; 2. previous exposure to antipsychotic agents; 3. current substance dependence; 4. deficits in general intelligence (IQ < 70); 5. neurological disorders or any medical condition; 6. clients not help-seeking or withdrawing their willingness to be followed by the service; 7. age range outside than 14–35.	CAARMS	Educational programmes in liaison with local health and non-health agencies who may encounter people potentially meeting the inclusion criteria. Mental health charities and voluntary organizations, local pastoral and educational services are also informed about the OASIS team. Thus, the educational programme is continuously ongoing and includes informal meetings, presentations and distribution of information materials. Information is also posted on a website and distributed in leaflets and newsletters.	Casemanagement, CBT, Medication (including low dose antipsychotica),
PACE	Mixed (longitudinal cohort and RCT)	referal	TAU vs AP-medication/CBT	1. 15-30 years old, 2. meeting the CHR criteria (APS, BLIPS, GRD)	1. presence of a current or past psychotic episode, 2. known organic cause for presentation, 3. past neuroleptic exposure equivalent to a total continuous haloperidol dose of more than 15 mg	CAARMS	na	A range of psychological, pharmacological, nutritional and multicomponent psychosocial interventions

PORT	Longitudinal cohort study	Referral/ self-referral	na	1. 14-29 years old, 2. Meeting the CHR criteria (APS, BLIPS, GRD)	1. the presence of a known organic disease of the central nervous system (CNS) such as epilepsy, 2. evidence of mental retardation and 3. a diagnosis of psychotic disorder according to ICD-10 criteria.	PANSS	In order to promote the programme, educational meetings and workshops for adolescents, teachers and parents were performed in high schools within the Lodz region, with the support of the Medical University of Lodz. Further training and workshops for psychiatrists and psychologists regarding the symptoms of ARMS has been provided by PORT team during local psychiatric conferences and meetings of the local section of the Polish Psychiatric Association.	CBT, diet supplementationwith omega-3 fatty acids andpharmacological treatment.
ROME	Observational cohort study	Referral	na	1. Meeting the CHR criteria, 2. Age 9-17	1. past or present psychosis, traumatic brain injury or any known neurological disorder, and 2. current drug or alcohol abuse. A history of drug use was permitted if symptoms had also been present in drug-free periods.	SIPS/SOPS	na	na
SAFE	Longitudinal cohort	Referral	na	1. Meeting the CHR criteria (APS, BLIPS, GRD), 2. Age 14-35 year	1. history of previous psychotic disorder or manic episode, 2. serious risk of suicide due to personality disorder, 3. substance abuse or addiction within 1 year of inclusion, 4. Known intellectual disability (IQ<70), neurological disorders, head injury or any other significant medical condition associated with psychiatric symptoms.	CAARMS	The launching of a website, dissimination leaflets, and provision telephone and email counseling in order to enlighten both experts and non-experts about psychosis intervention and to promote access to the clinic.	CBT oriented psychotherapy in an unstructured manner, antipsychotic medication
DUPS-U	Longitudinal cohort	Referral	na	1. Meeting the CHR criteria	1. past or present psychotic episode lasting longer than one week, 2. traumatic brain injury or any know neurological disorder, 3. verbal intellectual functioning <75	SIPS/SOPS	na	na

Abbreviations: APS: Attenuated Psychotic Symptoms, ARMS: At Risk Mental State, BLIPS: Brief Limited Intermitted Psychotic Symptoms, BPRS: Brief Psychotic Rating Scale, CAARMS: Comprehensive Assessment of At Risk Mental State, CBT: Cognitive Behavioral Therapy, CHR: Clinical High Risk, GRD: Genetic Risk and Deterioration, Na: not applicable, PANSS: Positive and Negative Syndrome Scale, SCID-I: Structured Clinical Interview of Diagnostic and Statistical Manual of Mental Disorders-I, SIPS/SOPS: Structured Interview of Prodromal Symptoms/Scale of Prodromal Symptoms, TAU: Treatment as Usual

SUPPLEMENT V

Bar plots of the frequency distribution of predicted survival of the survival groups.

Bar plots of the frequency distribution of the predicted and observed survival per survival group are presented for both 12 and 24 months. Groups with a high risk of survival implies a lower risk of transition to psychosis and vice versa. Equal distribution of predicted and observed survival per survival group indicates a well-discriminating model. The bar plot shows the percentage of the individual survival group, whereby the light gray denotes the predicted survival and the dark gray denotes the observed survival.

Figure 1a. ADAPT – 12 months

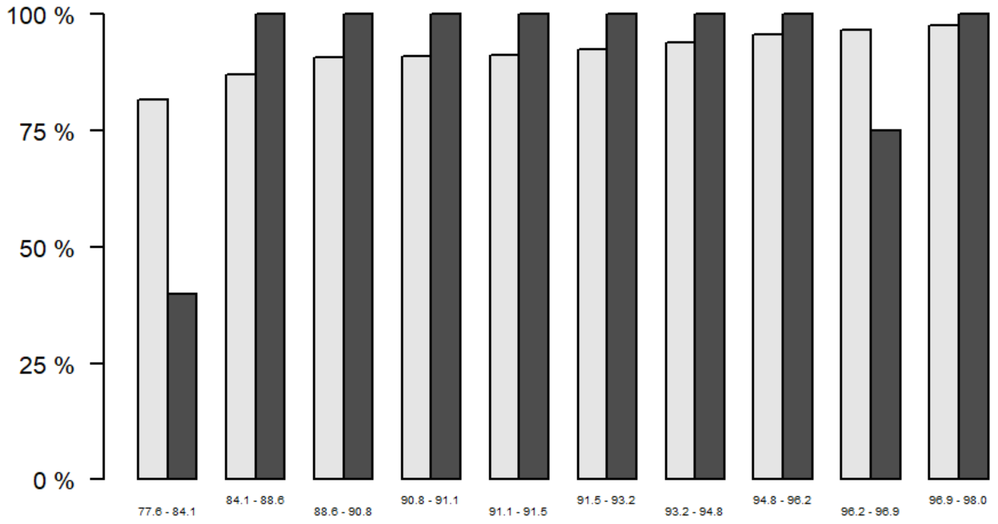

Survival group

Figure 1b. ADAPT – 24 months

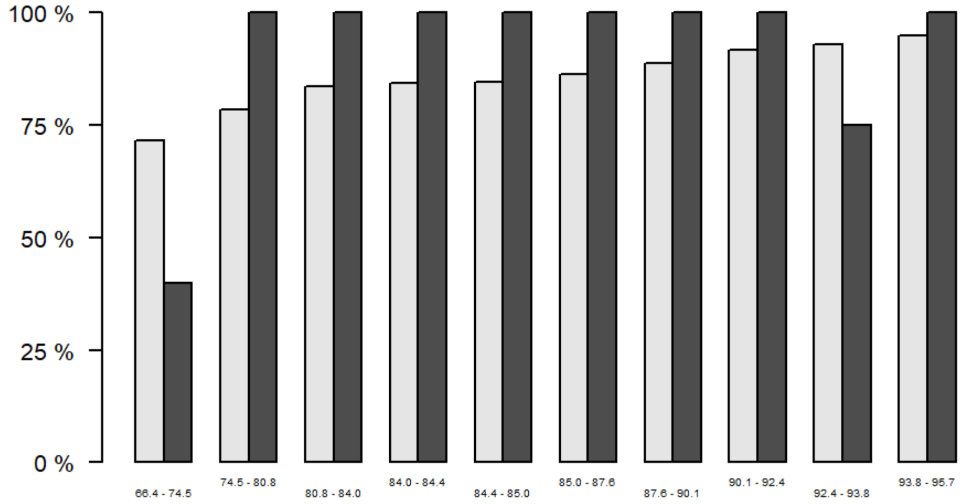

Survival group

Figure 2a. CAYR – 12 months

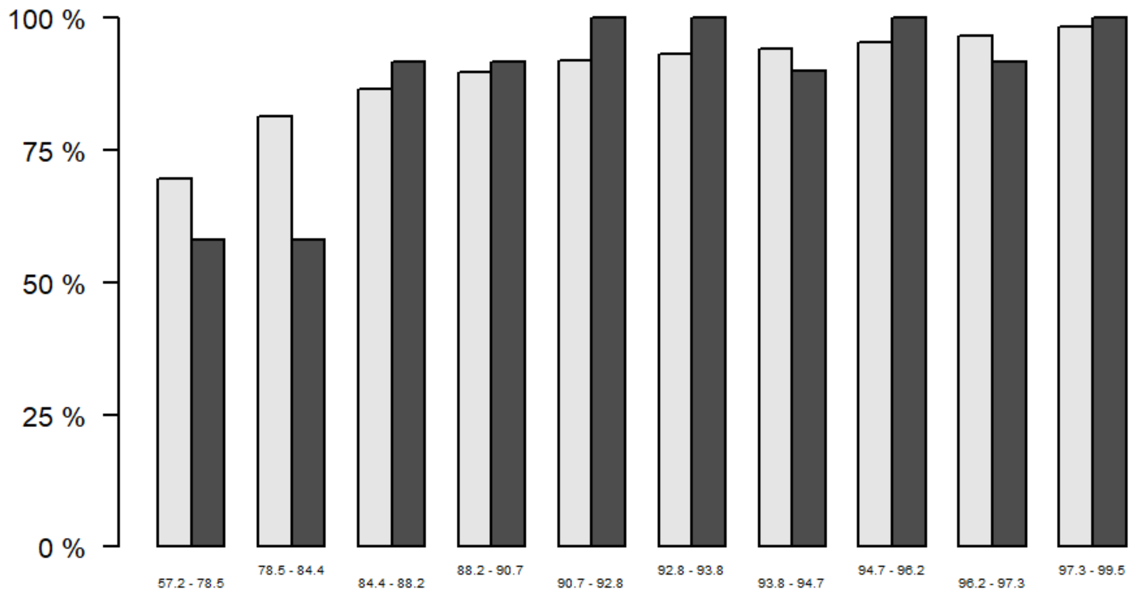

Figure 3a. DUPS-A – 12 months

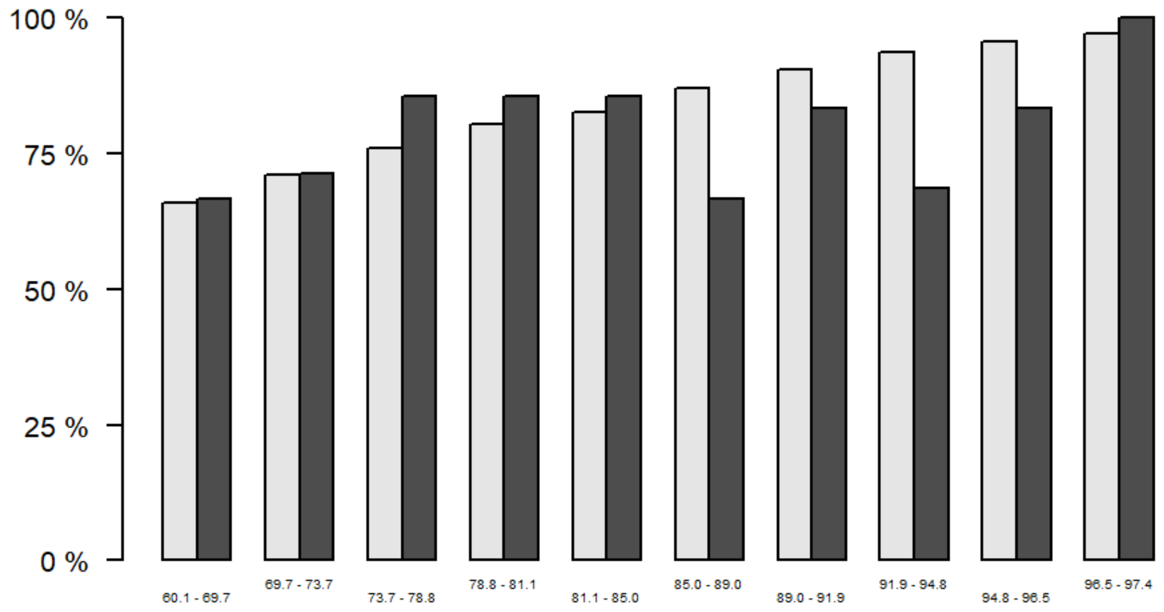

Figure 3b. DUPS-A – 24-months

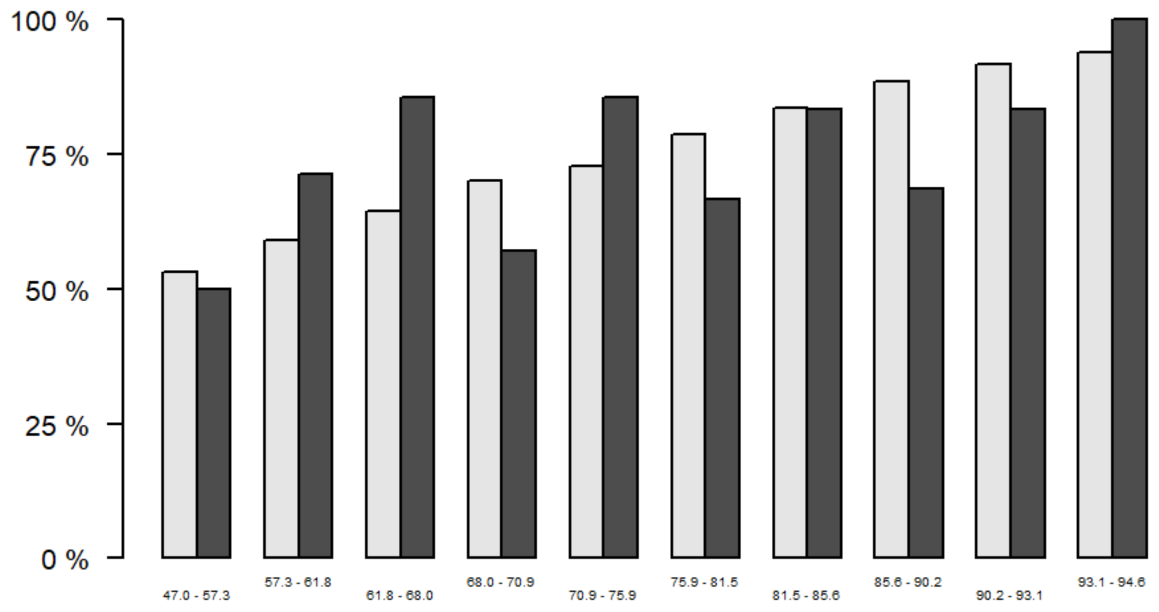

Figure 4. EDIE-NL – 12 months

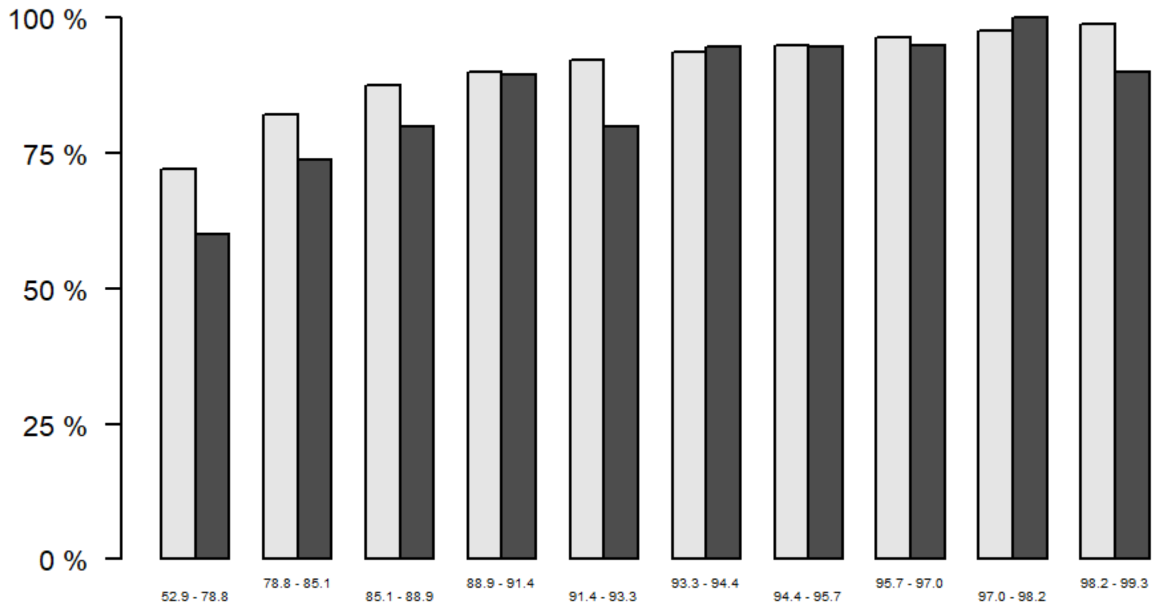

Figure 5a. EDIE-UK – 12 months

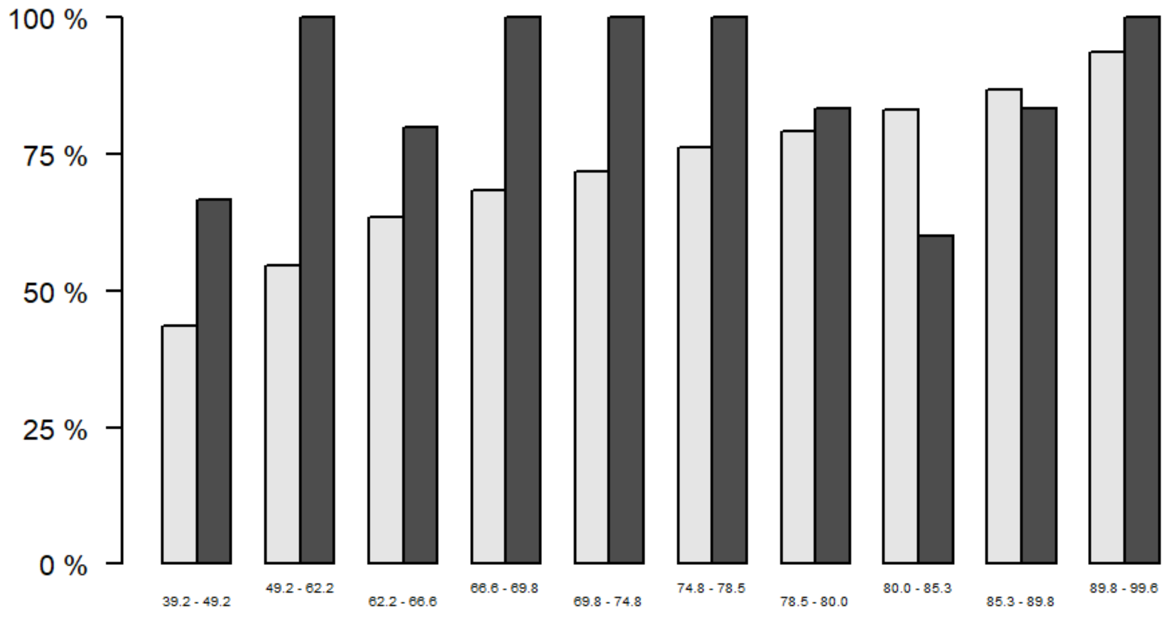

Figure 5b. EDIE-UK – 24 months

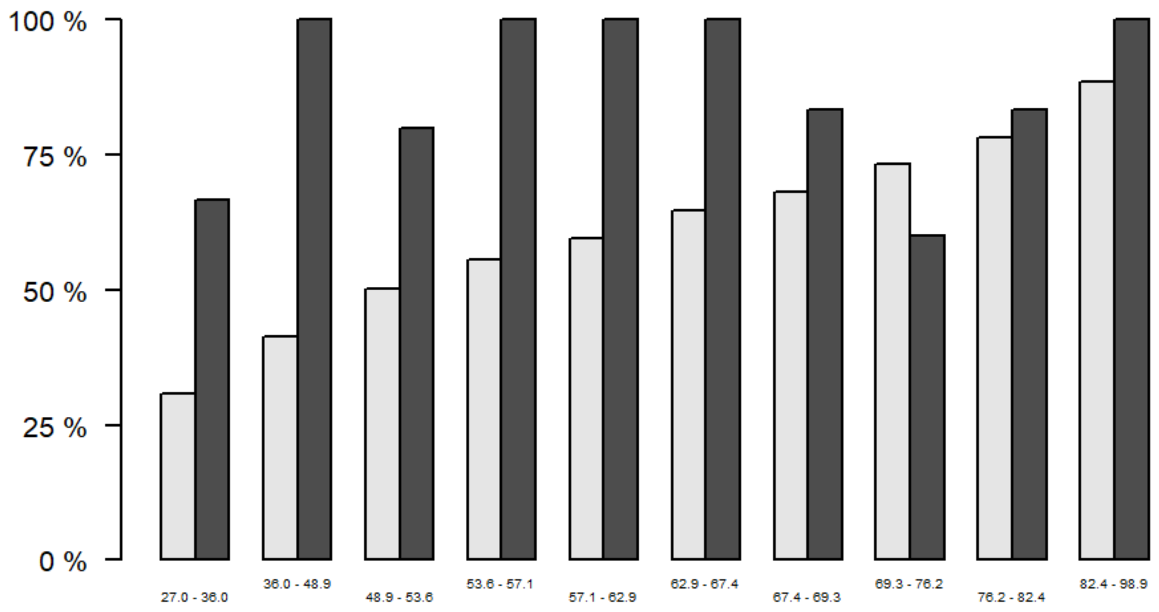

Figure 6a. FEPSY – 12 months

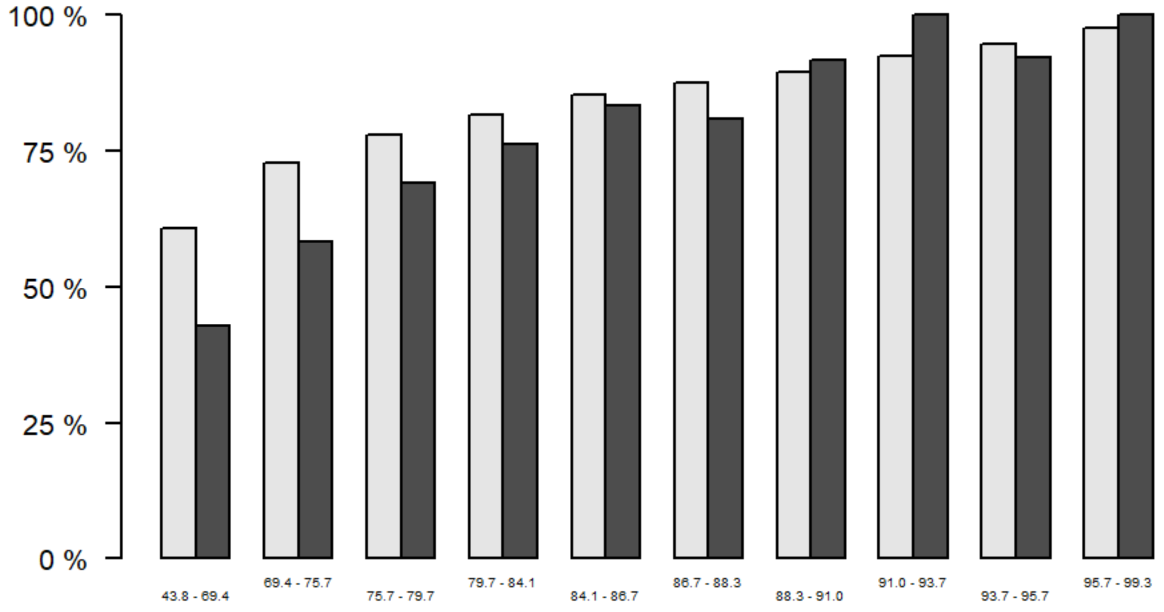

Figure 6b. FEPSY – 24 months

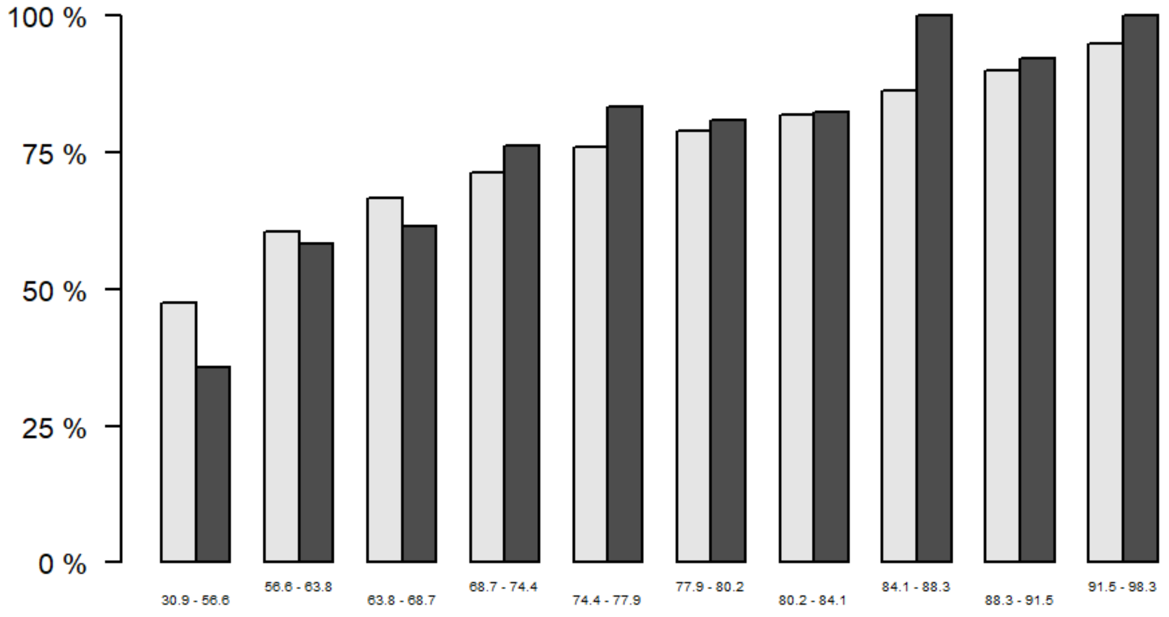

Figure 7a. FETZ – 12 months

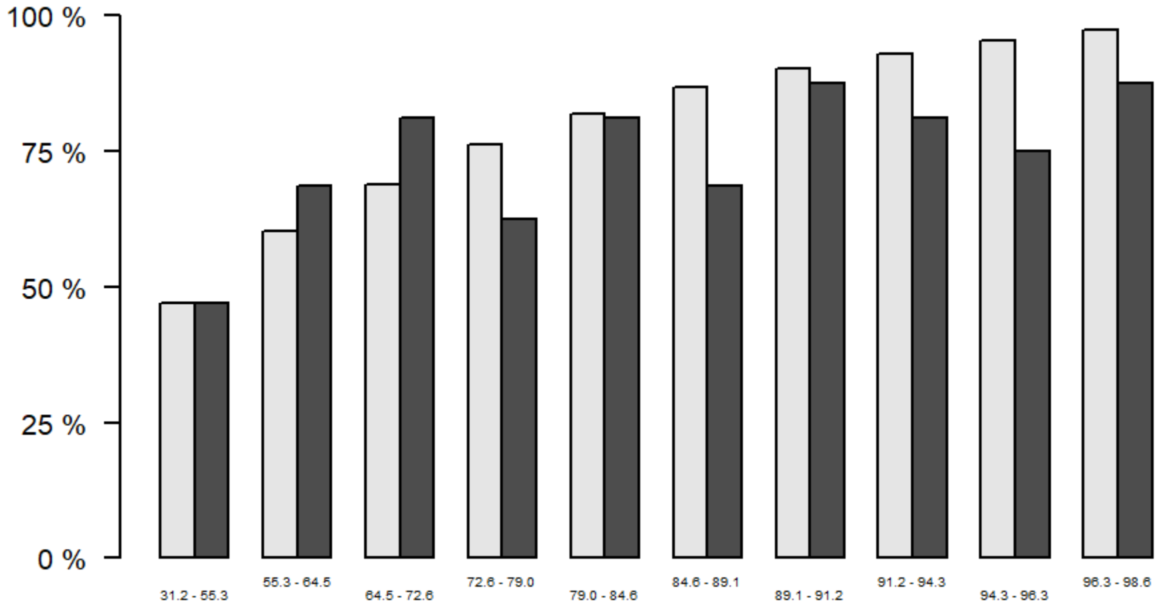

Figure 7b. FETZ – 24 months

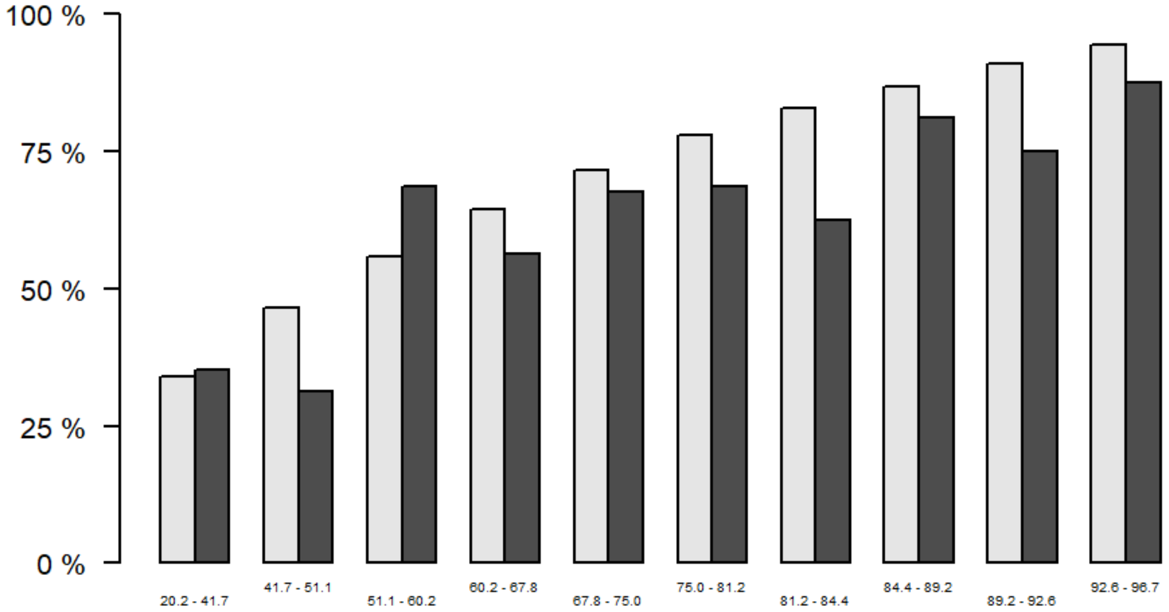

Figure 8a. GRAPE – 12 months

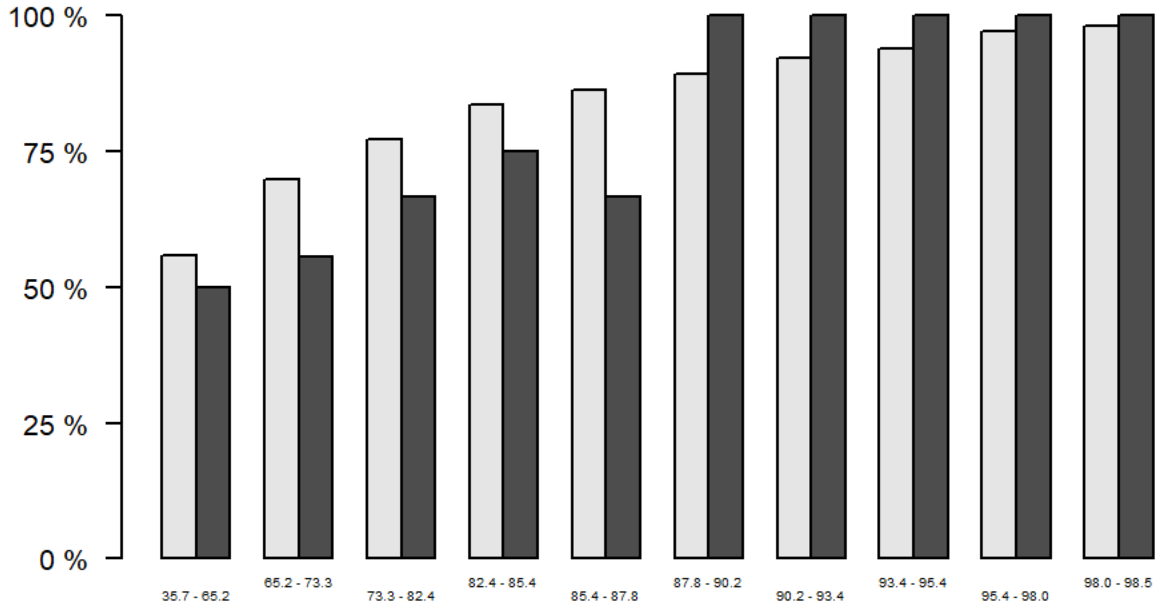

Figure 8b. GRAPE – 24 months

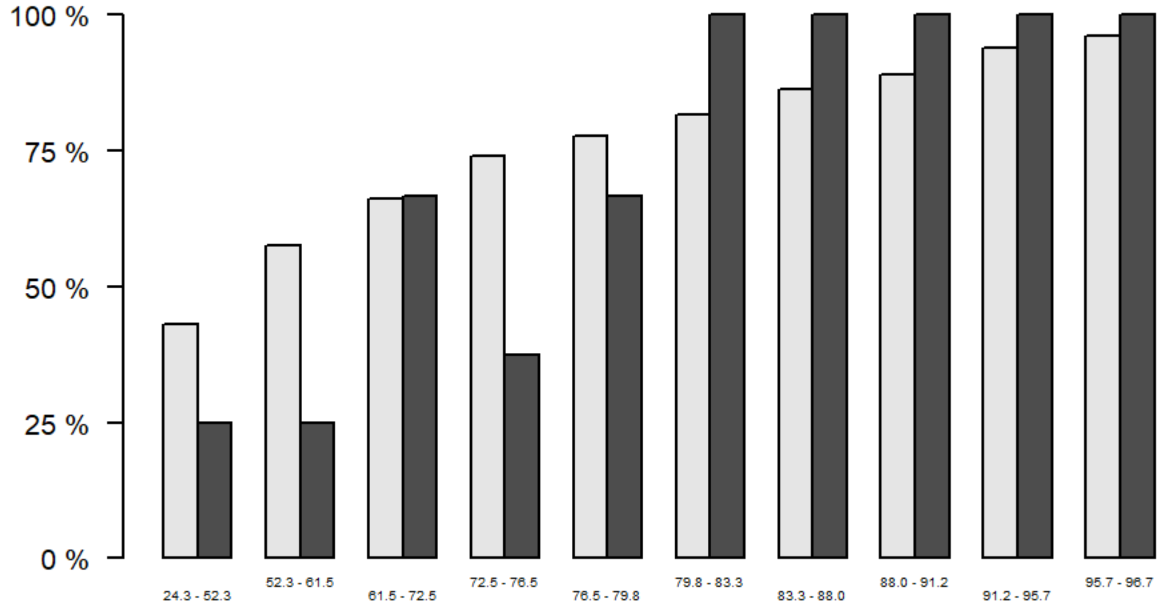

Figure 9a. INSTEP – 12 months

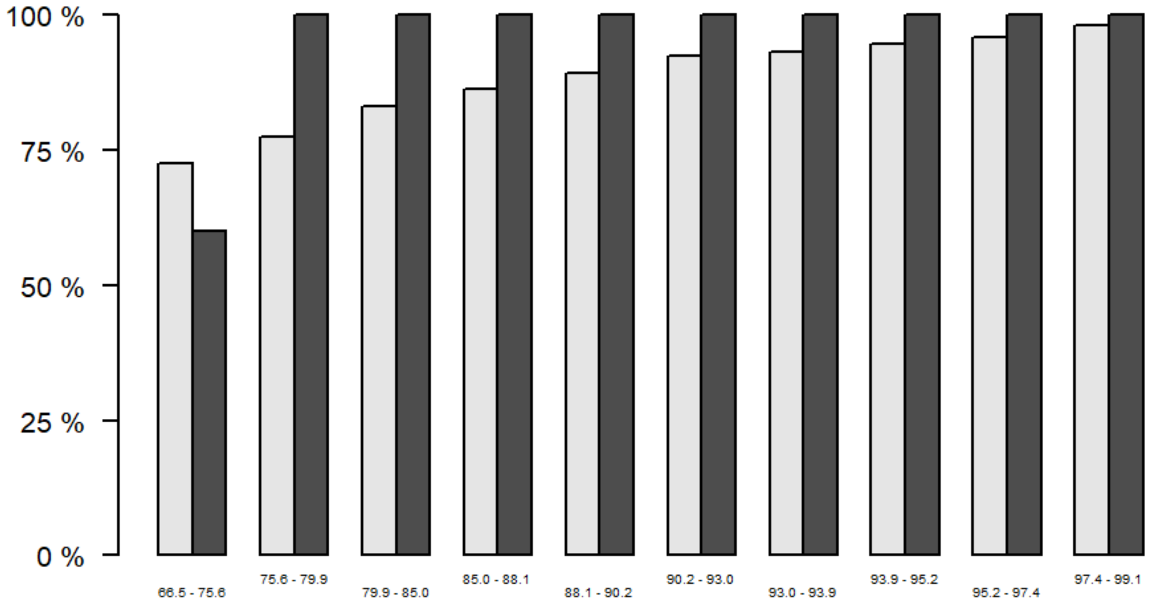

Figure 9b. INSTEP – 24 months

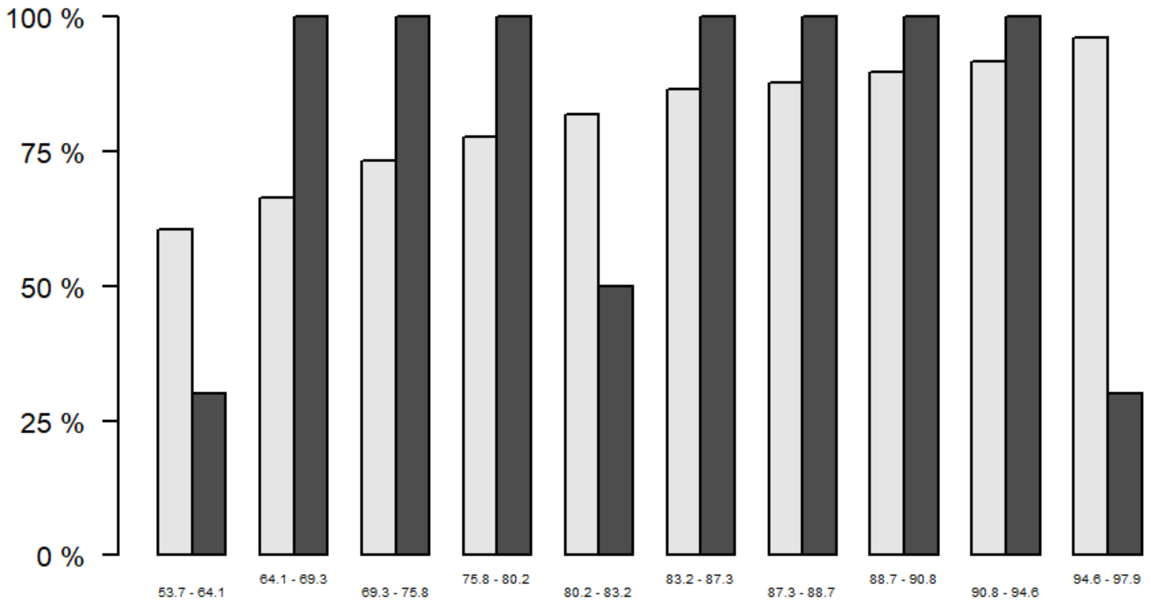

Figure 10a. OASIS – 12 months

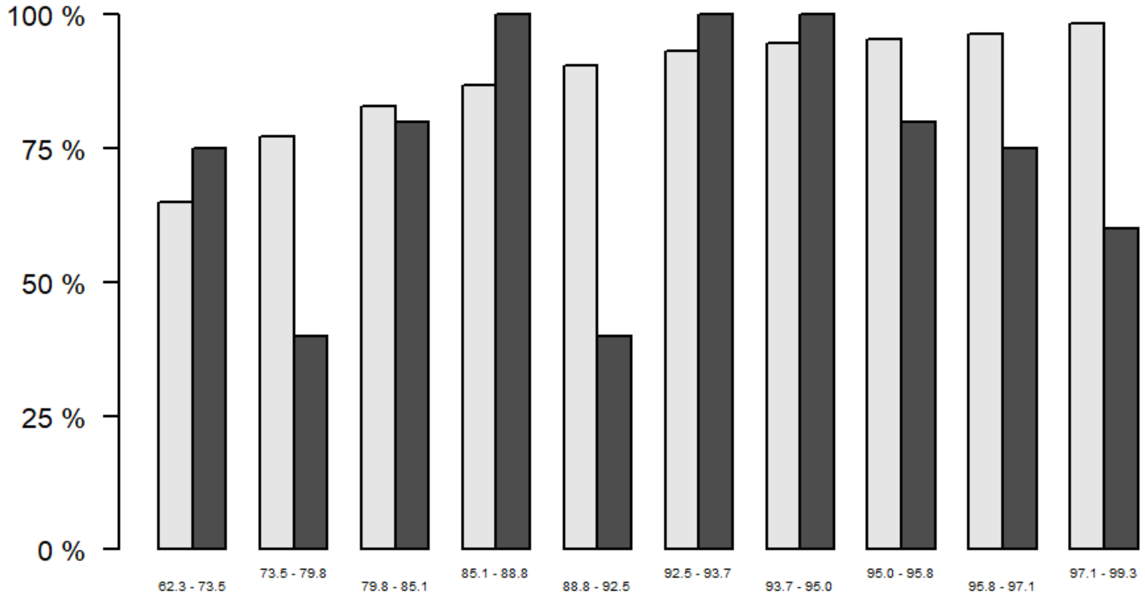

Figure 10b. OASIS – 24 months

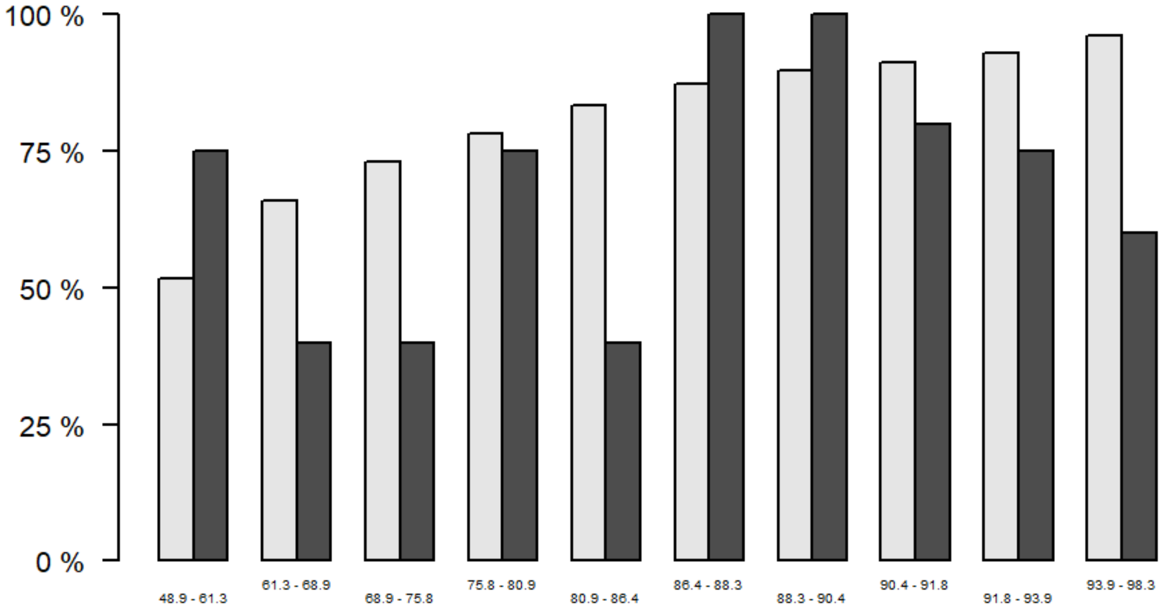

Figure 11a. PACE – 12 months

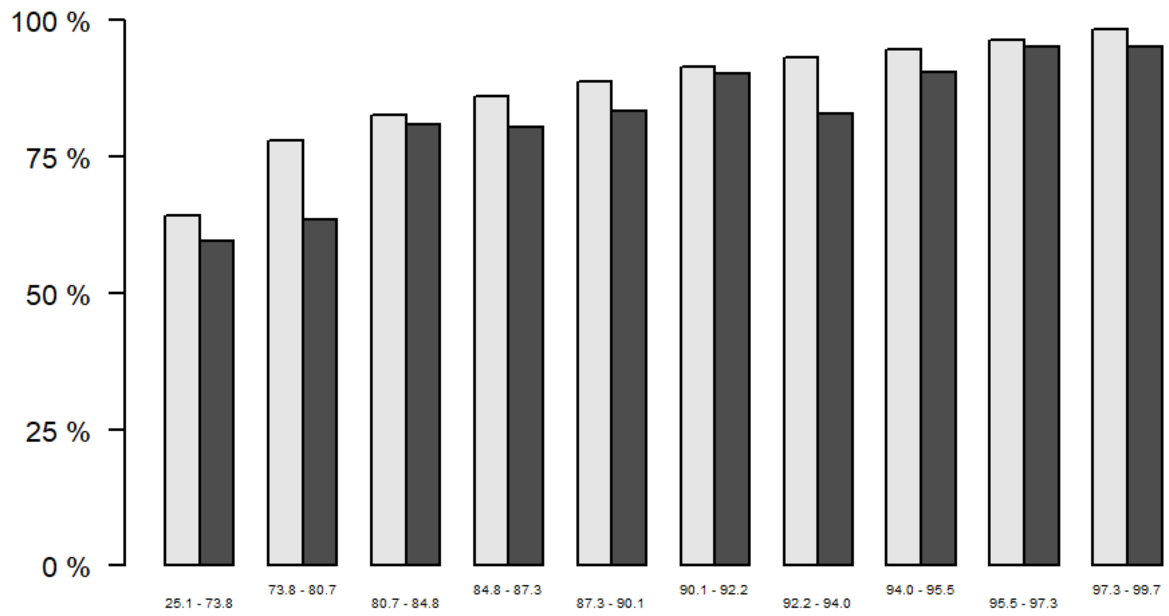

Figure 11b. PACE – 24 months

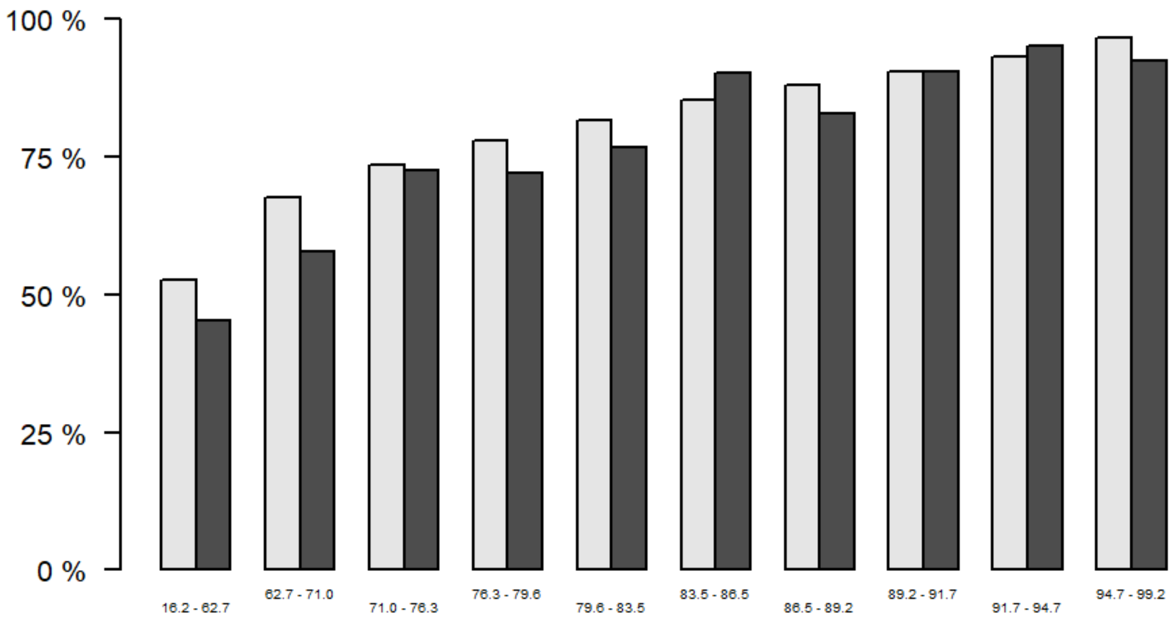

Figure 12a. PORT – 12 months

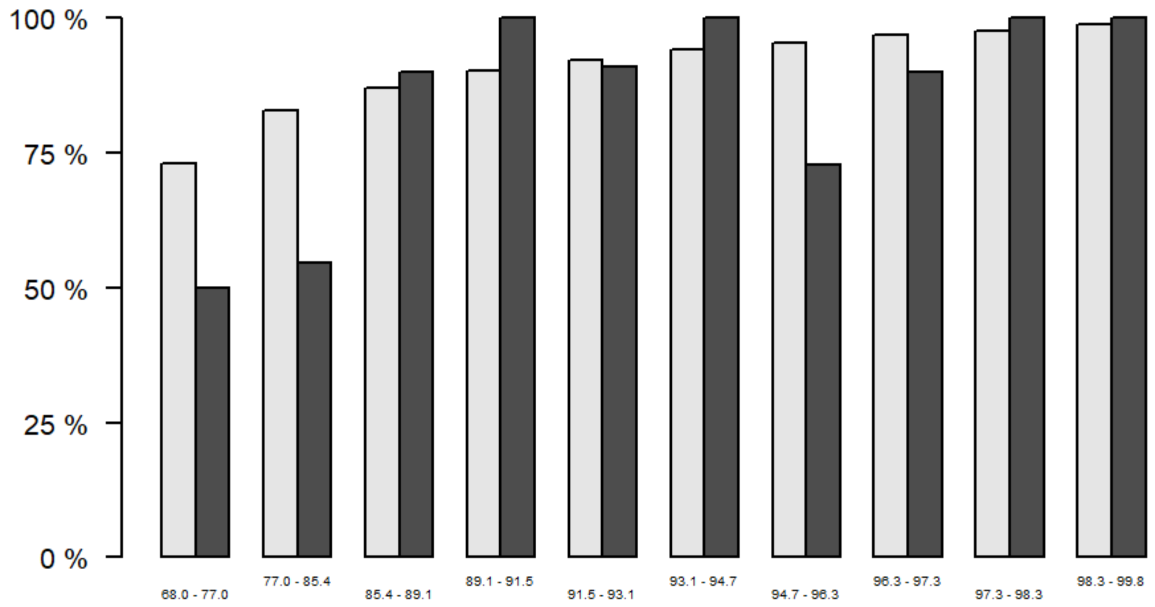

Figure 12b. PORT – 24 months

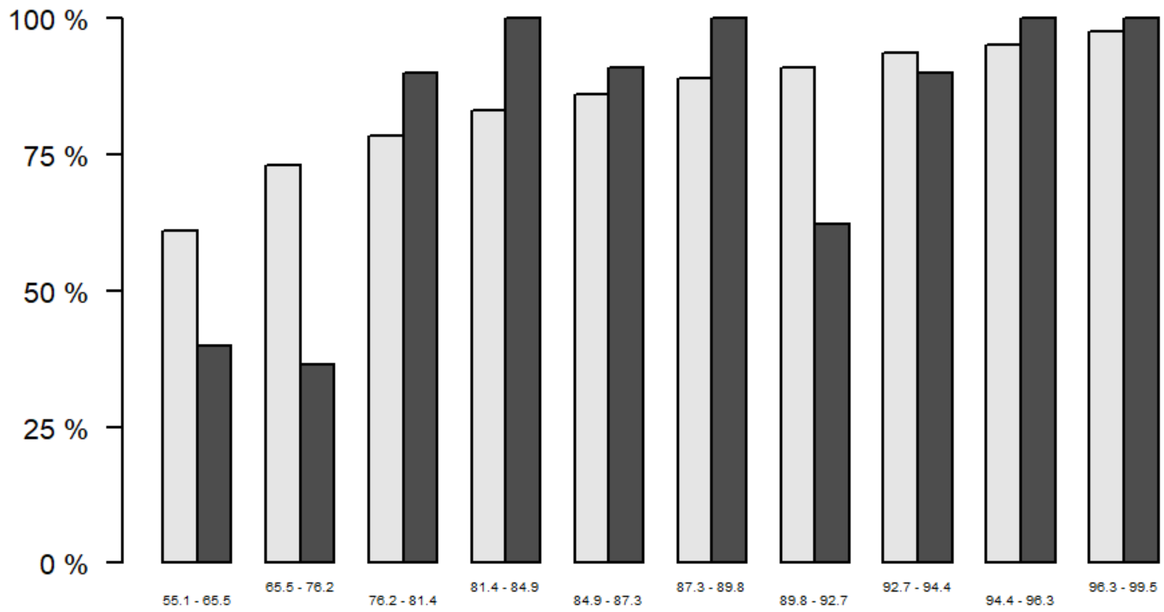

Figure 13a. ROME – 12 months

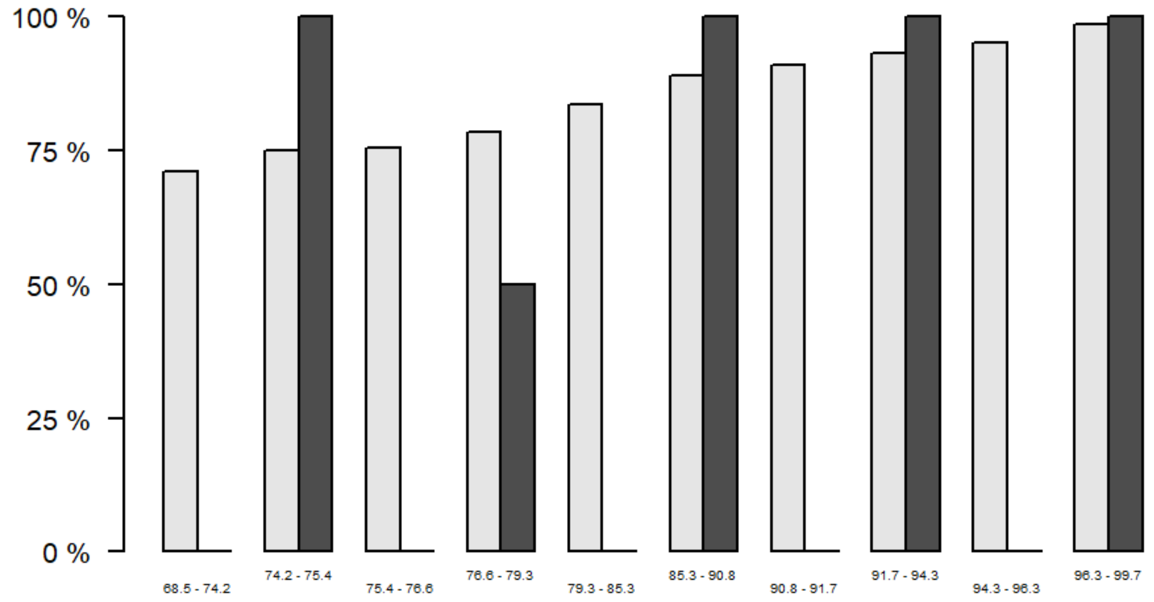

Figure 13b. ROME – 24 months

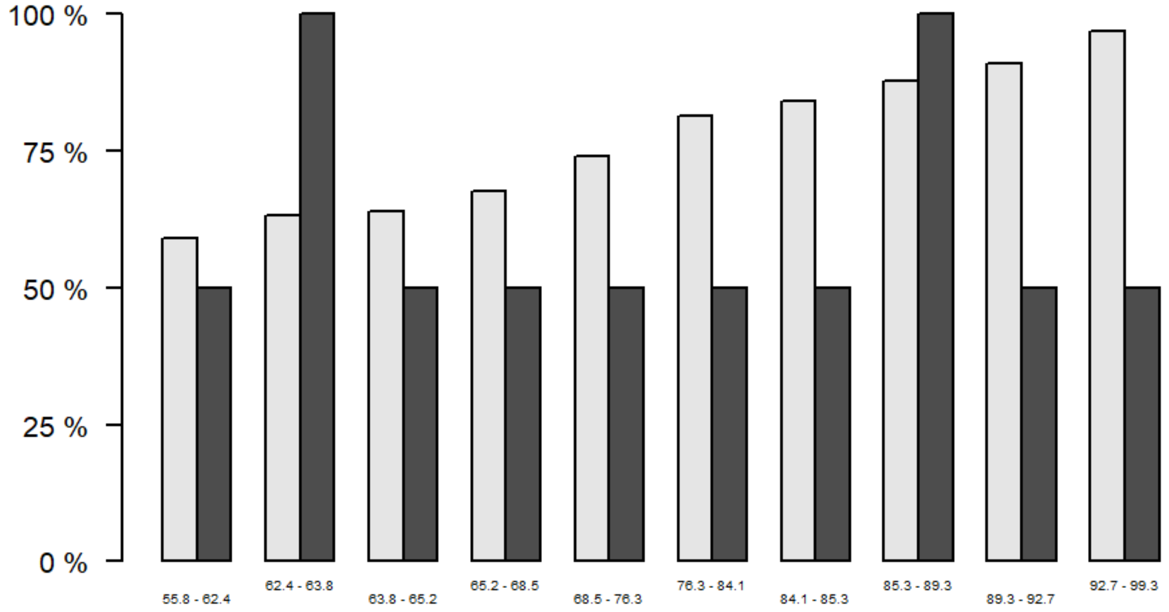

Figure 14a. SAFE – 12 months

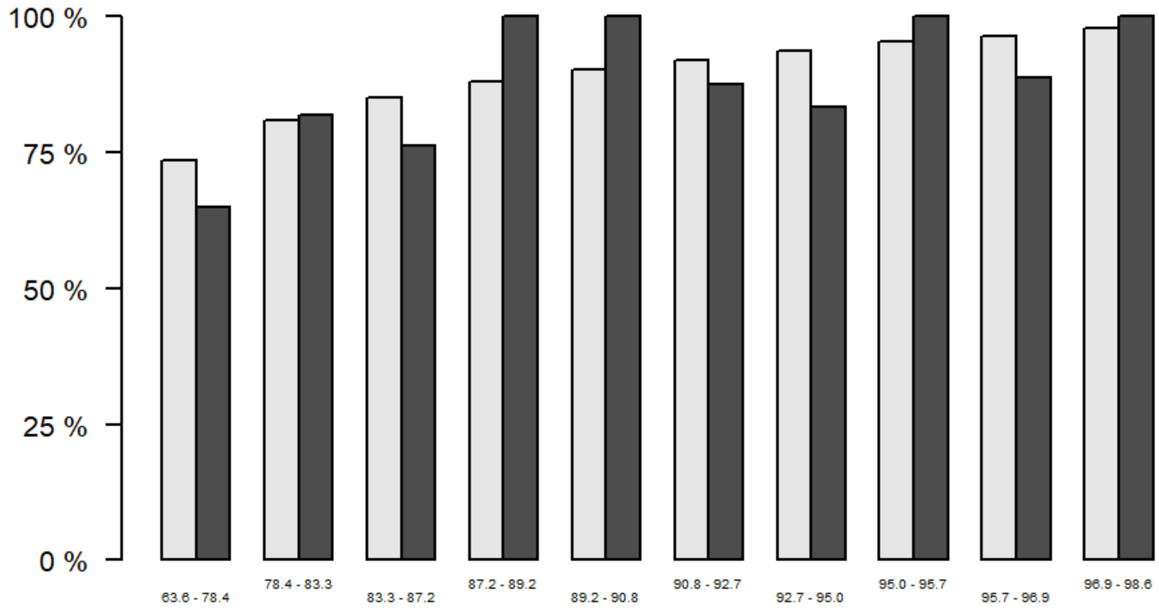

Figure 14b. SAFE – 24 months

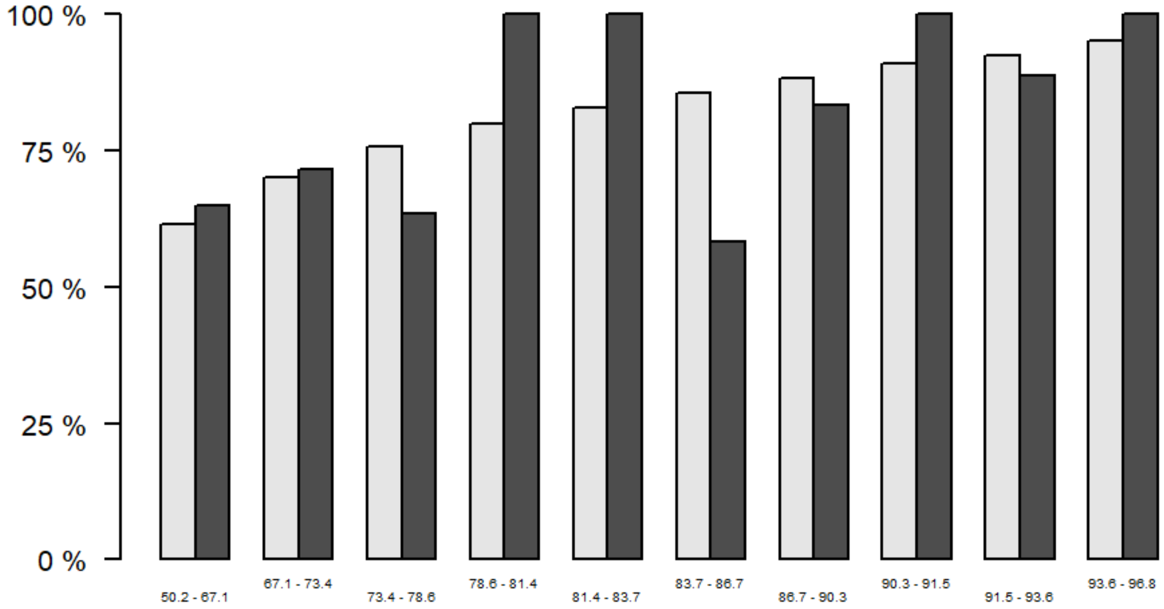

Figure 15a. DUPS-U – 12 months

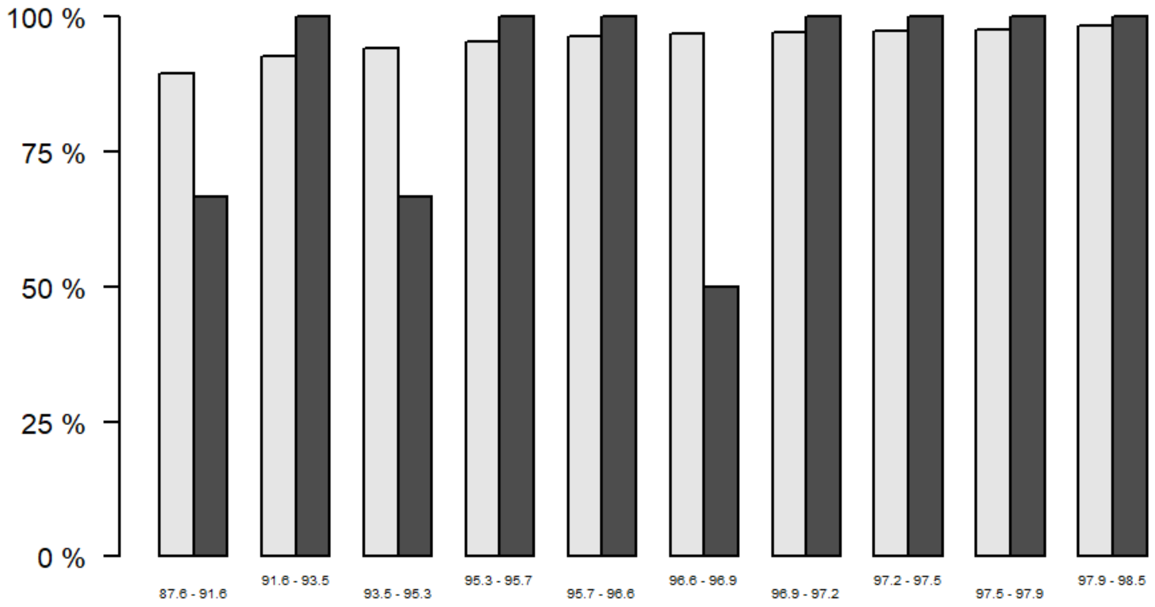

Figure 15b. DUPS-U – 24 months

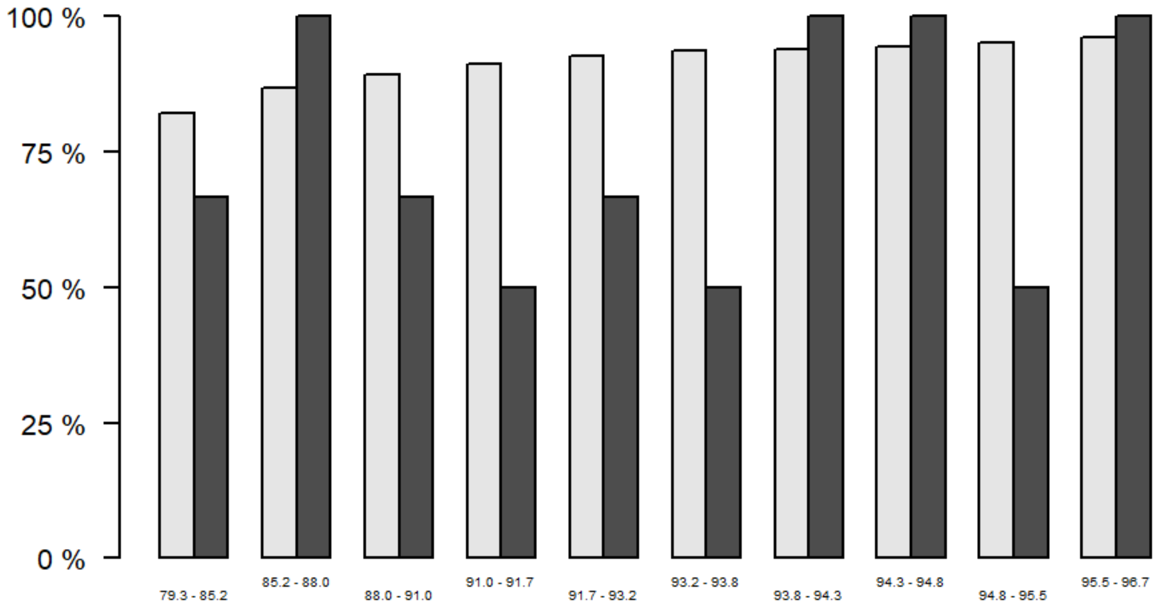

Supplement: Supplementary file 1 [file DataSheet_1.pdf]
